# Bacterial Membrane Vesicles as a Novel Strategy for Extrusion of Antimicrobial Bismuth Drug in Helicobacter pylori

**DOI:** 10.1128/mbio.01633-22

**Published:** 2022-09-26

**Authors:** Sumith Kumar, Christine Schmitt, Olivier Gorgette, Martial Marbouty, Magalie Duchateau, Quentin Giai Gianetto, Mariette Matondo, Jean-Michel Guigner, Hilde De Reuse

**Affiliations:** a Institut Pasteur, Département de Microbiologie, Unité Pathogenèse de *Helicobacter*, CNRS UMR 6047, Paris, France; b Institut Pasteur, Unité Technologie et Service Bio-lmagerie Ultrastructurale, Paris, France; c Institut Pasteur, Département de Génomes et Génétique, Unité Régulation Spatiale des Génomes, CNRS UMR 3525, Paris, France; d Institut Pasteur, Plateforme Protéomique, Unité de Spectrométrie de Masse pour la Biologie, C2RT, USR CNRS 2000, Paris, France; e Institut Pasteur, Hub de Bioinformatique et Biostatistique, Département de Biologie Computationelle, USR CNRS 3756, Paris, France; f IMPMC, Sorbonne University, CNRS UMR 7590, Museum National d'Histoire Naturelle, Paris, France; Rutgers University

**Keywords:** *Helicobacter pylori*, bismuth, membrane vesicles, antibiotic resistance, polyphosphate granules, chromosome conformation capture, Hi-C

## Abstract

Bacterial antibiotic resistance is a major threat to human health. A combination of antibiotics with metals is among the proposed alternative treatments. Only one such combination is successfully used in clinics; it associates antibiotics with the metal bismuth to treat infections by Helicobacter pylori. This bacterial pathogen colonizes the human stomach and is associated with gastric cancer, killing 800,000 individuals yearly. The effect of bismuth in H. pylori treatment is not well understood in particular for sublethal doses such as those measured in the plasma of treated patients. We addressed this question and observed that bismuth induces the formation of homogeneously sized membrane vesicles (MVs) with unique protein cargo content enriched in bismuth-binding proteins, as shown by quantitative proteomics. Purified MVs of bismuth-exposed bacteria were strongly enriched in bismuth as measured by inductively coupled plasma optical emission spectrometry (ICP-OES), unlike bacterial cells from which they originate. Thus, our results revealed a novel function of MVs in bismuth detoxification, where secreted MVs act as tool to discard bismuth from the bacteria. Bismuth also induces the formation of intracellular polyphosphate granules that are associated with changes in nucleoid structure. Nucleoid compaction in response to bismuth was established by immunogold electron microscopy and refined by the first chromosome conformation capture (Hi-C) analysis of H. pylori. Our results reveal that even low doses of bismuth induce profound changes in H. pylori physiology and highlight a novel defense mechanism that involves MV-mediated bismuth extrusion from the bacteria and a probable local DNA protective response where polyphosphate granules are associated with nucleoid compaction.

## INTRODUCTION

The discovery of antibiotics has revolutionized the treatment of bacterial infections. Antibiotics target various essential bacterial processes, generally leading to effective clearance of infection. However, their widespread use and increased misuse has led to high frequencies of antibiotic-resistant bacterial strains that make the existing antibiotics often ineffective (1). In addition to antibiotics, several alternatives such as bacteriophages, lysins, antimicrobial peptides, probiotics, and antibodies have been developed to treat bacterial infections (2). Several metals are known to efficiently kill bacteria, including most of the pathogens. Therefore, metal-based therapies in combination with antibiotics or alone have also been proposed to treat bacterial infections (3). However, there are only few examples of their use in clinics, since many metals are toxic to humans and side effects are common upon prolonged usage (4). Consequently, it is not easy to establish a defined dosage for effective therapy. Therefore, it is essential to understand the mechanisms of metal-related toxicity and how metals exert their antimicrobial properties, these processes being generally poorly defined.

In this work, we studied the action of the metal bismuth, which has been used since antiquity to treat gastric disorders and is, in many countries, still available as an over-the-counter drug under two forms, De-Nol (colloidal bismuth substrate) and Pepto-Bismol (bismuth subsalicylate). Recently, a therapy combining antibiotics, a proton pump inhibitor, and bismuth has been commercialized to treat Helicobacter pylori infections (5). H. pylori is a Gram-negative pathogen that infects the stomach of half of the human population worldwide. Infection by H. pylori is involved in various gastric pathologies such as gastritis, peptic ulcer, and gastric cancer, which kills about 800,000 individuals per year (6). The complex interplay of various cellular processes contributes to the pathogenesis of H. pylori, and thus it is essential to understand its complexity to successfully eradicate the infection (7). Since its discovery in 1982, H. pylori infection has been treated with antibiotics, but the increasing frequency of strains resistant to the restricted number of antibiotics available against H. pylori has strongly impaired the efficacy of the treatments (8). Lately, the WHO has recognized clarithromycin-resistant H. pylori as “high priority,” with a need to develop new treatments (9). Quadruple therapy involving bismuth along with two antibiotics (metronidazole and tetracycline) and a proton pump inhibitor is found to be more effective (84 to 97% eradication) than the “classical” combinatorial antibiotic therapy (60 to 67% eradication) (10, 11). The bismuth-based therapy is effective even against metronidazole-resistant strains (12), but the exact mechanism of action of bismuth in this treatment is not known. It is well established that H. pylori is killed by *in vitro* exposure to 10 to 20 μM concentrations of bismuth (13). Our recent studies showed that bismuth enters H. pylori via the Niu ABC nickel transporter and competes with nickel for uptake (14, 15). Upon uptake in H. pylori, bismuth binds to various metal-binding proteins, interferes with the metal metabolism, and inhibits various cellular processes, including essential functions (16). Very few articles, published more than 20 years ago, analyzed the consequences of bismuth exposure for H. pylori morphology and cellular integrity (17–19). The formation of dark electron-dense patches has been reported and was assigned to bismuth accumulation without any elemental composition validation. Thus, several questions remained open on the consequences of exposure to bismuth.

We reasoned that during treatment, H. pylori is exposed to high bismuth amounts only transitorily after ingestion and is unlikely to be in contact with high bismuth concentrations for long periods due to the low solubility, low absorption, and fast clearance of this metal in the stomach and also due to the variable accessibility of the infected gastric glands (10). Accordingly, low bismuth concentrations were measured in the blood plasma of quadruple-therapy-treated patients (10). Therefore, in the present study, we characterized for the first time the consequences of H. pylori exposure to sublethal amounts of bismuth and analyzed the bacterial response using a large panel of approaches, including electron microscopy, mass spectrometry (MS)-based proteomics, inductively coupled plasma optical emission spectrometry (ICP-OES), atomic spectroscopy, and chromosome conformation capture (Hi-C). Our study revealed that H. pylori has developed original protective strategies to respond to bismuth exposure. We observed that H. pylori extrudes bismuth by membrane vesicles (MVs). In addition, we defined the chemical nature of bismuth-induced electron-dense patches to be polyphosphate (poly-P) granules that are associated with DNA compaction. Our study provides a novel view on both defense and protection mechanisms of a major bacterial pathogen against a toxic metal used in clinics.

## RESULTS

### Bismuth subcitrate minimal inhibitory concentration determination for Helicobacter pylori strain G27.

Bismuth salts, including bismuth subcitrate (CBS) used in the anti-H. pylori treatment, are insoluble in water. In previous studies, colloidal suspensions of CBS at pH 7.0 were used as a standard solution to test antimicrobial effects against H. pylori (13). However, under these conditions, active CBS concentrations and thus available bismuth amounts are variable due to the colloidal and precipitation-prone nature of CBS. We circumvented this issue by preparing a soluble concentrated stock of bismuth (1.2 M) in 10% liquid ammonia (pH 10.0), which we diluted extemporaneously in the test medium for minimal inhibitory concentration (MIC) determination. Unlike the colloidal solution, our soluble stock was stable at room temperature and gave highly reproducible MIC values. Using these conditions, we consistently measured a CBS MIC of 6.0 μg mL^−1^ (15 μM) for H. pylori strain G27. The sublethal dose of bismuth that we chose for this study was 2.5 μM bismuth (0.17× MIC), a concentration at which growth of H. pylori is not affected under microaerophilic conditions (see Fig. S1A in the supplemental material).

10.1128/mbio.01633-22.1 FIG S1 (A) Effect of sublethal bismuth concentration on growth of H. pylori strain G27. Bacterial growth was monitored in Brucella liquid medium supplemented with 10% FCS. Bismuth was added at a final concentration of 2.5 μM at zero time point. Bacterial optical density was measured at different time points. (B and C) Color and TEM analysis of purified membrane vesicles of H. pylori treated with bismuth and controls. (B) Preparations of MVs from bismuth-treated bacteria are brownish-black, while nontreated control MVs are translucent. (C) Quantification of purified MV size distribution. Diameter of MVs was determined by a dynamic light scattering assay. For each sample, three independent data acquisitions were done. The assay was repeated 2 times with biological duplicates. The intensity-weighted distribution of MVs was plotted against the diameter size. Size distributions between MVs of control and bismuth-treated samples were compared using the Kolmogorov-Smirnov statistical test. ****, *P* < 0.0001. (D and E) TEM analysis of purified MVs from control bacteria (D) and from bismuth-treated bacteria (E). Download FIG S1, PDF file, 1.6 MB. Copyright © 2022 Kumar et al. 2022 Kumar et al. https://creativecommons.org/licenses/by/4.0/ This content is distributed under the terms of the Creative Commons Attribution 4.0 International license.

### MVs of bismuth-treated H. pylori cells.

**FIG 1:**
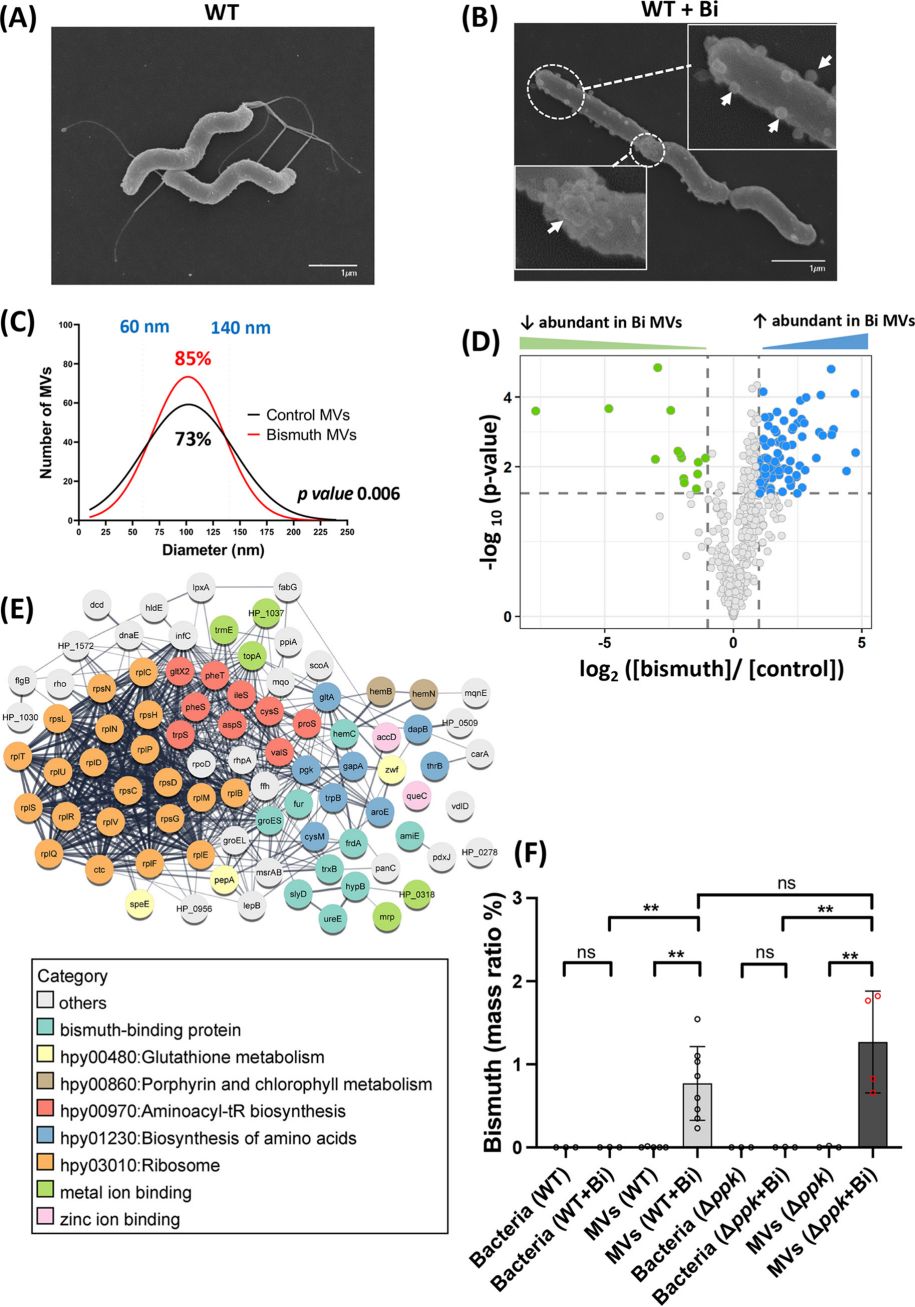
Characterization of MVs from bismuth-treated and untreated H. pylori cells. (A and B) Scanning electron microscopy (SEM) of control bacteria (A) and bismuth-treated bacteria (2.5 μM, 6 h) (B). Surface-attached MVs are visible in the bismuth-treated sample, indicated by white arrows in the insets. (C) Size distribution of MVs purified from control and bismuth-treated H. pylori cells. MV diameter was measured with the ImageJ software using TEM images of a total of 600 MVs for each condition, and the Gaussian equation was fitted to the histogram data to obtain a frequency distribution plot. The nonparametric Kolmogorov-Smirnov test shows statistically significant differences between the two distributions (*P* = 0.006). (D) Volcano plot representing the differential abundance of proteins purified from MVs from bismuth-treated H. pylori cells and from control bacteria. The *x* axis shows the bismuth/control fold change value in logarithmic scale. The *y* axis shows the −log_10_ of the adjusted *P* value showing statistical significance. The horizontal dashed line corresponds to a *P* of 0.01 used as the threshold cutoff. Vertical dotted lines correspond to log_2_ fold changes of ±2. Blue dots (87 proteins) and green dots (14 proteins) represent more abundant and less abundant proteins, respectively, in MVs of bismuth-treated sample. (E) STRING network analysis of differentially abundant proteins present in the vesicles of bismuth-treated bacteria. The thickness of the gray lines represents the strength of the interaction between proteins. The functional category of each protein is indicated by the color code. (F) ICP-OES metal content of purified MVs and their parent H. pylori cells exposed or not to bismuth. Wild-type (WT) bacteria and a mutant carrying a deletion of the *ppk* gene (Δ*ppk*) were analyzed. The amount of bismuth is expressed as a mass ratio percentage of dry mass (*n* ≥ 3). The limit of detection, 10 μg/L, corresponds to 10 ppb. Statistics using the Mann-Whitney test are shown. **, *P* < 0.01; ns, nonsignificant.

The effect of bismuth exposure on H. pylori morphology was assayed by growing bacteria in the presence of sublethal bismuth concentrations. Scanning electron microscopy (SEM) on fixed control bacterial samples revealed the characteristic helical shape with an intact smooth cell surface (Fig. 1A). Bismuth-treated bacteria retained the helical shape but presented round surface-attached MVs. While some of these MVs could be detected at the surface of nontreated cells, their amount per cell in bismuth-exposed cultures was reproducibly higher (Fig. 1B, white arrows). These MVs were of variable size and appeared throughout the surface of the bismuth-treated bacteria (Fig. 1B). Due to the nature of SEM images, a global quantification of surface-attached MV structures was not possible (Fig. 1B). However, the increased appearance of MVs on bismuth-treated H. pylori prompted us to better characterize these MVs.

### Bismuth treatment results in the release of a homogeneous MV population.

To better understand the impact of bismuth exposure on H. pylori vesicle production, we purified the soluble MVs from H. pylori cultures exposed or not exposed to bismuth. The MV purification yields (particles/mL culture) were similar under the two conditions. When compared to that of an untreated culture, the MV pellet of bismuth-treated cultures was found to be brown-black in nature (Fig. S1B). Purified MVs were then visualized by transmission electron microscopy (TEM) to determine their size distribution and to rule out bacterial contamination of the purified sample (Fig. S1D and E). The size of the MVs was measured using ImageJ software, and a normal size distribution with mean sizes of 103 and 100 nm, respectively, was observed under the two conditions, with and without bismuth treatment (Fig. 1C). However, the global size distributions of MVs from the two populations were slightly different: untreated samples exhibited a slightly wider size distribution and were more heterogeneous, with diameters ranging from 20 nm to 240 nm (Fig. 1C). In contrast, MVs purified from bismuth-treated samples displayed a slightly more homogeneous size distribution, ranging from 40 nm to 210 nm. Eighty-five percent of MVs from bismuth-treated bacteria had a diameter between 60 and 140 nm, compared to 73% for untreated bacteria (Fig. 1C).

We also verified the differences in population size distribution of purified MVs using dynamic light scattering (DLS) and observed a similar distribution shift in the population of MVs in response to bismuth (Fig. S1C). Together, these results demonstrate that sublethal bismuth treatment affects the color and size distribution of the released MVs along with an increased amount of vesicle structures attached to the bacterial surface.

### Exposure of H. pylori cells to bismuth causes secretion of MVs with a specific protein cargo.

We next asked whether the shift toward a more homogeneous size distribution of bismuth-exposed H. pylori MVs was accompanied by a differential protein cargo content. A global quantitative proteomic analysis was carried out to compare the MV populations from cells exposed or not exposed to bismuth (*n* = 3) (Table S1). In MVs isolated from bismuth-treated bacteria, a total of 486 proteins was identified, representing 32% of the reference proteome of strain G27 (20), compared to 455 proteins in MVs from untreated bacteria. A total of 32 proteins were exclusively detected in MVs from bismuth-treated bacteria, including several previously identified bismuth-binding H. pylori proteins, such as AmiE, HypB, TrxA, and HP0958 (21), along with proteins involved in metal transport, metal binding, and metabolic enzymes (Table S1). On the other hand, 14 proteins that are exclusively absent in MVs from bismuth-exposed bacteria include metabolic enzymes and uncharacterized proteins (Table S1).

10.1128/mbio.01633-22.8 TABLE S1 (A) Proteins present and absent in the MVs originating from bismuth-treated bacteria compared to those from untreated bacteria. (B) Differentially abundant proteins present in MVs of bismuth-treated and nontreated samples. Download Table S1, DOCX file, 0.03 MB. Copyright © 2022 Kumar et al. 2022 Kumar et al. https://creativecommons.org/licenses/by/4.0/ This content is distributed under the terms of the Creative Commons Attribution 4.0 International license.

Differentially abundant proteins in the two samples are represented as a volcano plot in Fig. 1D. A total of 101 proteins were present in significantly different amounts in the two MV populations (Fig. 1D; Table S1). Eighty-seven proteins were enriched (log_2_ fold change > 2, adjusted *P* value < 0.01) in the MVs from bismuth-treated bacteria, while only 14 proteins were enriched in the vesicles from untreated bacteria (Table S1). SlyD, a bismuth-binding protein previously characterized in our lab (22), was found to be 2.7-log_2_ fold more abundant in MVs of bismuth-treated bacteria. To establish whether SlyD is indeed induced by the exposure to bismuth and to test whether we could validate the enrichment of SlyD in the MVs of bismuth-treated bacteria, we carried out reverse transcription real time quantitative PCR (RT-qPCR) and Western blot analysis. The *slyD* transcript is upregulated by 2-log_2_ fold in bismuth-treated samples compared to untreated bacteria (Fig. S2A). Western blot analysis of total bacterial lysates using a specific anti-SlyD antibody revealed a 2-fold-increased amount of SlyD in the bismuth-exposed bacteria compared to nonexposed cells (Fig. S2B). Western blot analysis of soluble fractions from purified MVs further validated our mass spectrometry data, as larger amounts of SlyD (2.4-fold the amount of that in control MVs) are observed in the MVs originating from bismuth-treated bacteria (Fig. S2B). Thus, bismuth enters the bacteria and induces the expression of the *slyD* gene, and this results in increased protein levels of SlyD in the cell and its subsequent accumulation in MVs.

10.1128/mbio.01633-22.2 FIG S2 Expression analysis of the H. pylori SlyD protein in response to bismuth treatment in cell lysates and soluble MV fractions. (A) RT-qPCR analysis of the *slyD* transcript on control wild-type or Bi-treated samples. (B) Western blot analysis of the levels of SlyD protein using anti-SlyD antibodies on lysates of bacteria exposed to bismuth or on controls and on the corresponding soluble MV fractions. Stain-free images were used as a loading control. Download FIG S2, PDF file, 0.2 MB. Copyright © 2022 Kumar et al. 2022 Kumar et al. https://creativecommons.org/licenses/by/4.0/ This content is distributed under the terms of the Creative Commons Attribution 4.0 International license.

Gene ontology (GO) analysis and KEGG pathway enrichment of proteins significantly more abundant in the MVs of bismuth-treated bacteria highlight various biological processes (Fig. 1E). With bismuth, enrichment was observed in ribosomal proteins (hpy03010; 21 proteins; enrichment *P* value = 4.3e−07), zinc ion binding proteins (GO:0008270; 9 proteins; *P* value = 0.043), and heme binding proteins (GO:0020037; 3 proteins; *P* value = 0.088). Biological pathways were also enriched, including aminoacyl tRNA biosynthesis (hpy00970; 9 proteins; *P* = 0.026), glutathione metabolism (hpy00480; 3 proteins; *P* = 0.042), and biosynthesis of amino acids (hpy01230; 8 proteins; *P* = 0.095). In addition, we identified several bismuth-binding proteins as well as other metal-binding proteins among these proteins (Fig. 1E; Table S1). Among the metal binding proteins, the amount of Fur, an iron-responsive transcriptional regulator, was strongly increased. Since the *fur* gene is negatively autoregulated in response to iron, we postulate that the observed upregulation of Fur could be due to its inactivation by the binding of bismuth. These data highlight an overall impact of bismuth exposure on protein abundance and subsequent enrichment in MVs, some of which might be due to transcriptional deregulation associated with Fur inactivation.

For the 14 proteins less abundant in the MVs from bismuth-treated bacteria, they include different metal transporters, such as the cation transporter CadA (Ni^2+^ homeostasis), the HbpA periplasmic dipeptide/heme-binding protein, and FrpB2, a TonB-dependent outer membrane transporter predicted to be involved in hemoglobin and thus iron acquisition (Table S1). Recently, the expression of *frpB2* has been shown to be regulated by nickel and involved in the uptake of this metal (Table S1) (23). This suggests downregulation of metal uptake systems upon bismuth exposure. Collectively, these results provide a global view of the changes in the protein content of MVs upon bismuth exposure. Notably, our results provide evidence for the first time that a metal ion such as bismuth affects the size distribution and cargo content of secreted MVs in H. pylori.

### Bismuth is concentrated in MVs produced by bismuth-treated H. pylori cells.

To further characterize the consequences of bismuth exposure for H. pylori, we used ICP-OES to measure the amount of this metal in whole bacteria and in purified MVs from H. pylori cultures treated with bismuth (2.5 μM) or not (Table S2). To avoid contamination by bismuth, vesicles were purified by density gradient centrifugation using Optiprep. As expected, no bismuth was detected in the nontreated control MVs or in the bacteria (Fig. 1F). Most interestingly, the MVs purified from bismuth-exposed cultures displayed a highly significant 0.4% bismuth mass ratio per dry weight of the purified vesicles. The purified MVs still displayed the brownish color mentioned above, confirming that bismuth was indeed responsible for this color. To exclude the possibility that bismuth precipitated from the medium might contribute to the metal measured in MVs, we measured bismuth after the same purification procedure but in the absence of bacteria. No bismuth was measured under this condition, just like in the untreated sample. In contrast, the bismuth-treated bacteria from which these MVs originated did not contain detectable amounts of this metal. Our results demonstrate that bismuth is accumulated in bismuth-exposed MVs (Fig. 1F; Table S2).

10.1128/mbio.01633-22.9 TABLE S2 Measurement of bismuth levels in H. pylori cellular lysates and purified MVs. Bismuth concentration was determined by measuring the emission spectra at three different wavelengths. Subscript values under λ correspond to the emission signals observed at different wavelengths (mean average of biological and technical replicates). The limit of detection is 10 μg/L (10 ppb). Mass ratio (%) was calculated by dividing the mass of bismuth detected in the sample by the total mass of the sample used for analysis. WT, wild type. Download Table S2, DOCX file, 0.01 MB. Copyright © 2022 Kumar et al. 2022 Kumar et al. https://creativecommons.org/licenses/by/4.0/ This content is distributed under the terms of the Creative Commons Attribution 4.0 International license.

Overall, these data suggest that selective enrichment of multiple bismuth-binding proteins in MVs from bismuth-treated bacteria plays a crucial role in transporting or accumulating bismuth into MVs. Also, the specific presence of bismuth in the MVs and not in the parent bacteria from which the MVs were secreted suggests that H. pylori utilizes MVs to escape bismuth-related toxicity by “packaging” this metal into the MVs and releasing it in the extracellular environment.

### Bismuth exposure induces the formation of electron-dense patches in the H. pylori cytoplasm.

**FIG 2:**
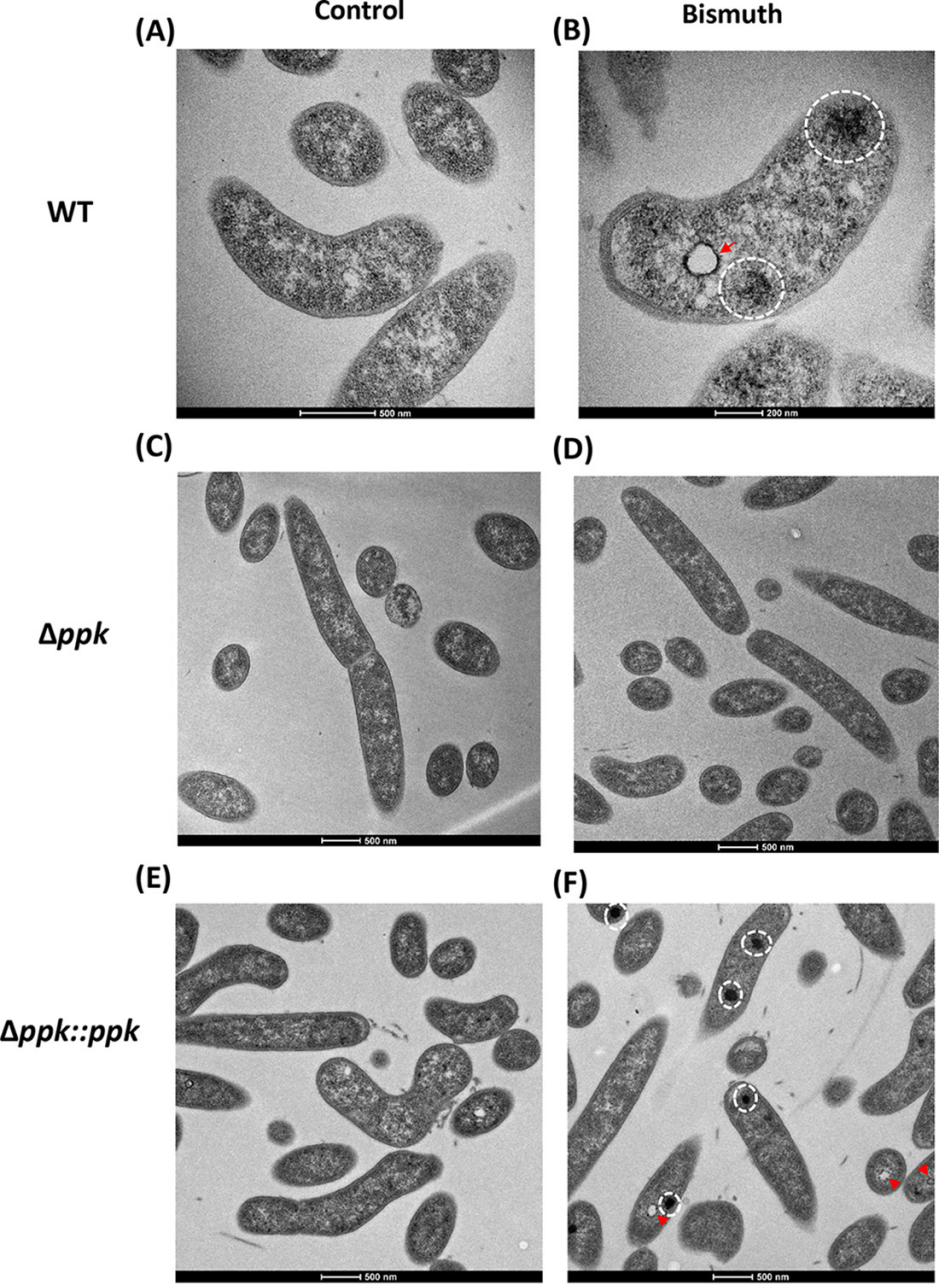
Transmission electron microscopy analysis of bismuth-treated H. pylori cells. (A) Untreated control bacteria exhibit a uniform membrane structure with an intact cytoplasm. (B) Bismuth-treated bacteria show an enrichment in electron-dense cytoplasmic structures (highlighted by white circles) as well as “vacuole-like” structures surrounded by electron-dense structures (red arrow). (C) Untreated Δ*ppk* mutant cells. (D) Bismuth-treated Δ*ppk* mutant cells, where no electron-dense structures are observed. (E) Untreated Δ*ppk* mutant complemented with a wild-type *ppk* copy. (F) Bismuth-treated Δ*ppk* mutant complemented with a wild-type *ppk* copy. In this strain, electron-dense structures are restored upon bismuth treatment (white circles and red arrowheads).

In addition to defining the effects on H. pylori morphology and cell surface, we analyzed the intracellular consequences of bismuth exposure by TEM. For TEM analysis, 60-nm thin slices were prepared and stained with or without uranyl acetate and lead citrate. With these methods, H. pylori cells conserved intact membrane structures (Fig. 2A). In contrast to untreated samples that presented uniform periplasmic and cytoplasmic regions, a 7-h-long sublethal bismuth treatment resulted in the formation of electron-dense regions in the H. pylori cytoplasm (Fig. 2B; Fig. S3A). As a control to rule out precipitation of lead citrate or uranyl acetate in the bacterial sections, TEM analysis was performed on unstained sections and revealed the same electron-dense regions (Fig. S3C and D).

10.1128/mbio.01633-22.3 FIG S3 TEM analysis of bismuth-treated H. pylori cells and of controls. (A) A low-magnification electron micrograph highlights the abundance of H. pylori bismuth-treated sections with electron-dense regions; some of them are indicated by red arrows. (B) Quantification of electron-dense regions in control and bismuth-treated bacteria; a total of 360 control bacterial sections and 278 sections from bismuth-treated sample were analyzed. The number of electron-dense regions per section was plotted against the percentage of bacterial sections. (C and D) Control TEM of bacterial sections without uranyl acetate and lead citrate staining for nontreated bacterial sections (C) and for bismuth-treated bacterial sections (D). The presence of electron-dense regions and of “vacuole-like” structures surrounded by an electron-dense region, visible only in panel D, is highlighted by white open circles with a red arrow in the inset. Download FIG S3, PDF file, 2.3 MB. Copyright © 2022 Kumar et al. 2022 Kumar et al. https://creativecommons.org/licenses/by/4.0/ This content is distributed under the terms of the Creative Commons Attribution 4.0 International license.

The distribution of these electron-dense regions was quantified. As much as 41% of the bacterial sections (*n* = 278) from bismuth-treated cells present at least one electron-dense region in the cytoplasm, and 4% have more than one such region (Fig. S3B). However, given that these sections correspond to only a 60-nm thin section, it is probable that almost every cell in the bismuth-treated samples contains at least one such region. Sections from a control sample (*n* = 360) showed essentially no electron-dense regions (Fig. S3B). In addition to the dense regions, we also observed, upon bismuth treatment, the formation of white “vacuole-like” circular structures with an electron-dense periphery (Fig. 2B; Fig. S3D, red arrows). These vacuole-like structures were frequently present along with the diffused electron-dense region in the bacterial cytoplasm of bismuth-treated samples, as seen in the low-magnification image in Fig. S3A. These electron-dense structures are most likely a consequence of the intracellular action of bismuth and are compatible with a global alteration in cellular physiology.

### Energy-dispersive X-ray analysis of the H. pylori response to bismuth.

**FIG 3:**
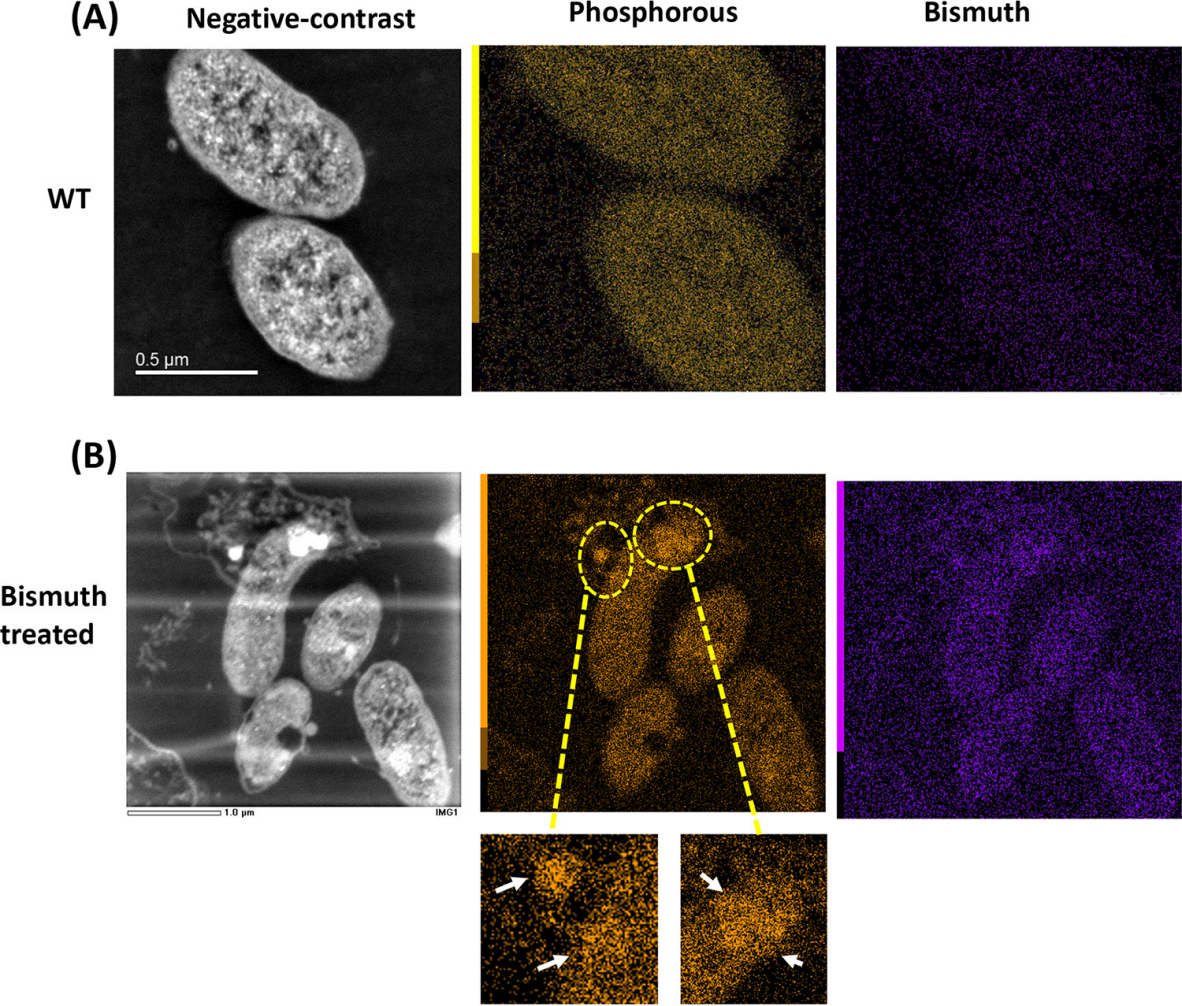
Elemental mapping of bismuth-treated H. pylori cells. (A) Scanning-transmission electron micrographs (STEM) of untreated bacteria. The negative-contrast image of control bacterial sections is shown together with the EDX map for phosphorus and bismuth. (B) STEM negative-contrast image of H. pylori cells exposed to bismuth. Electron-dense regions are seen as bright white regions. EDX maps of phosphorus and bismuth are shown. Accumulation of phosphorus in the cytoplasm of these bismuth-treated bacteria is highlighted by yellow open circles and white arrows in the inset panels; this accumulation overlaps with the electron-dense regions.

We next tested the chemical nature of the intracellular electron-dense structures induced by bismuth. We performed elemental mapping analysis of bacterial sections using TEM coupled with energy-dispersive X-ray (EDX). First, EDX analysis of the control H. pylori cell ultrathin sections using a focused beam over the bacterial cytoplasm revealed a uniform distribution of various elements in the bacterial section (Fig. S4A). We took spectra for phosphorus, calcium, nickel, oxygen, sulfur, magnesium, and bismuth. Surprisingly, a similar analysis of the bismuth-treated samples revealed that the electron-dense regions induced by bismuth were not enriched in bismuth but displayed a strong accumulation of phosphorus (Fig. S4B). The EDX analysis was, however, limited to specific focused regions of the bacterial section (Fig. S4B). To get an overall elemental composition of the complete bacterial sections, scanning transmission electron microscopy (STEM) analysis coupled with EDX was carried out. As above, the control samples exhibited a uniform distribution of different elements (Fig. 3A). In bismuth-treated bacteria, the accumulation of electron-dense regions was visible in the STEM images (Fig. 3B, white regions). The overall elemental analysis again showed that these regions present little or no enrichment in bismuth but rather a specific accumulation of phosphorus (Fig. 3B). These phosphorus-rich structures varied largely, from diffuse to highly concentrated circular regions (Fig. 3B, white arrows). These data indicate that H. pylori sublethal exposure to bismuth alters the elemental profile and favors the formation of phosphorus-rich electron-dense regions.

10.1128/mbio.01633-22.4 FIG S4 TEM and EDX microanalysis of bismuth-treated and control H. pylori cells. (A) TEM image of a control untreated bacterial section, with dotted lines showing the region where the electron beam was focused for EDX analysis. Elemental composition by EDX spectra shows a uniform distribution of elements with no selective enrichment. (B) TEM image of a bismuth-treated bacterium exhibiting an electron-dense structure, with a dotted circle and lines showing the region where the electron beam was focused for EDX analysis. Elemental composition by EDX spectra revealed selective enrichment of phosphorus in the analyzed region. A red circle highlights the peak of phosphorus. Download FIG S4, PDF file, 1.9 MB. Copyright © 2022 Kumar et al. 2022 Kumar et al. https://creativecommons.org/licenses/by/4.0/ This content is distributed under the terms of the Creative Commons Attribution 4.0 International license.

### Bismuth induces accumulation of poly-P granules in H. pylori.

The phosphorus composition of the electron-dense structures induced by bismuth in H. pylori is reminiscent of poly-P granules. The formation of poly-P granules, composed of linear chains of more than 10 orthophosphate (P_i_) molecules under a high-energy polymeric form, is a general stress response in bacteria and has been shown to occur in response to anaerobic and nutrient stress in H. pylori (24). Poly-P granules are polymerized by the activity of polyphosphate kinase (PPK) encoded by the *ppk* gene, which was previously characterized in H. pylori (25, 26). To test whether the electron-dense regions induced by bismuth could correspond to poly-P granules, we constructed an H. pylori *ppk* deletion mutant and a complemented *Δppk* strain with an active copy of the *ppk* gene at the native locus. Thin-section electron microscopy showed that upon bismuth exposure, the *ppk* deletion prevents the appearance of phosphorus-rich electron-dense regions, which was recovered in the complemented strain (Fig. 2C to F). This demonstrates that the electron-dense phosphate-rich regions induced by bismuth in the H. pylori cytoplasm correspond to poly-P granules. The presence of poly-P granules could not be assessed in MVs because of their small size. However, we asked whether poly-P granules could mediate the export or accumulation of bismuth into MVs. Bismuth measurement by ICP-OES showed that MVs from the Δ*ppk* mutant concentrate bismuth similarly to the wild-type MVs (Fig. 1F). This strongly suggests that export of bismuth into MVs is independent of poly-P granules. Finally, we tested the role of PPK in H. pylori resistance to toxic doses of bismuth and found that the MIC for the *Δppk* mutant was comparable to that for the wild-type strain. These data show that poly-P accumulation is a response to bismuth that does not provide H. pylori with resistance to its toxicity.

### Response of PPK to bismuth and its requirement for proper cell segregation.

In bacteria, poly-P accumulation depends on the balance between its synthesis by PPK and its hydrolysis by the PPX exopolyphosphatase. The PPX protein has also been characterized in H. pylori (27). In our G27 strain, the *ppx* gene appears to be essential, since a deletion mutant could not be constructed. The transcriptional response of *ppk* and *ppx* to 2.5 μM bismuth was measured (Fig. S5A and B). No significant induction of *ppk* transcription was found by bismuth. We constructed a strain expressing a FLAG-tagged PPK protein from its native locus. By Western blot analysis, slightly increased PPK protein amounts (1.2-fold compared to those of controls) were repeatedly observed in response to bismuth (Fig. S5C). Bismuth-mediated upregulation of PPK protein and possibly of its activity might be responsible for the increased occurrence of poly-P granules in the cytoplasm. Moreover, expression of *ppx* was increased 2.3-fold in the Δ*ppk* mutant, suggesting a *ppk*-mediated suppression of *ppx* expression (Fig. S5B). However, *ppx* expression in the wild-type background was not regulated by bismuth, but in the Δ*ppk* mutant, bismuth inhibited expression of *ppx* (Fig. S5B). Our results highlight the critical regulation of *ppx* and *ppk* and the interplay of bismuth in modulating the expression via induction of poly-P granules.

10.1128/mbio.01633-22.5 FIG S5 Expression analysis of *ppk* and *ppx* transcripts in response to bismuth treatment in different genetic backgrounds of H. pylori strain G27. Differential fold change of the *ppk* transcript level (A) and of the *ppx* transcript level (B) measured by RT-qPCR (*n* = 3). A one-way ANOVA test was done to compute statistical significance. ****, *P* < 0.0001; ***, *P* < 0.001. (C) Western blot analysis of total extracts of a wild-type strain expressing a FLAG-tagged PPK protein that was either bismuth-treated (WT Bi) or nontreated (WT); the Δ*ppk* strain serves as a negative control. The PPK-FLAG protein is shown by a red arrow. The stain-free image of the same gel (shown below) was used for calibration. Download FIG S5, PDF file, 0.2 MB. Copyright © 2022 Kumar et al. 2022 Kumar et al. https://creativecommons.org/licenses/by/4.0/ This content is distributed under the terms of the Creative Commons Attribution 4.0 International license.

**FIG 4:**
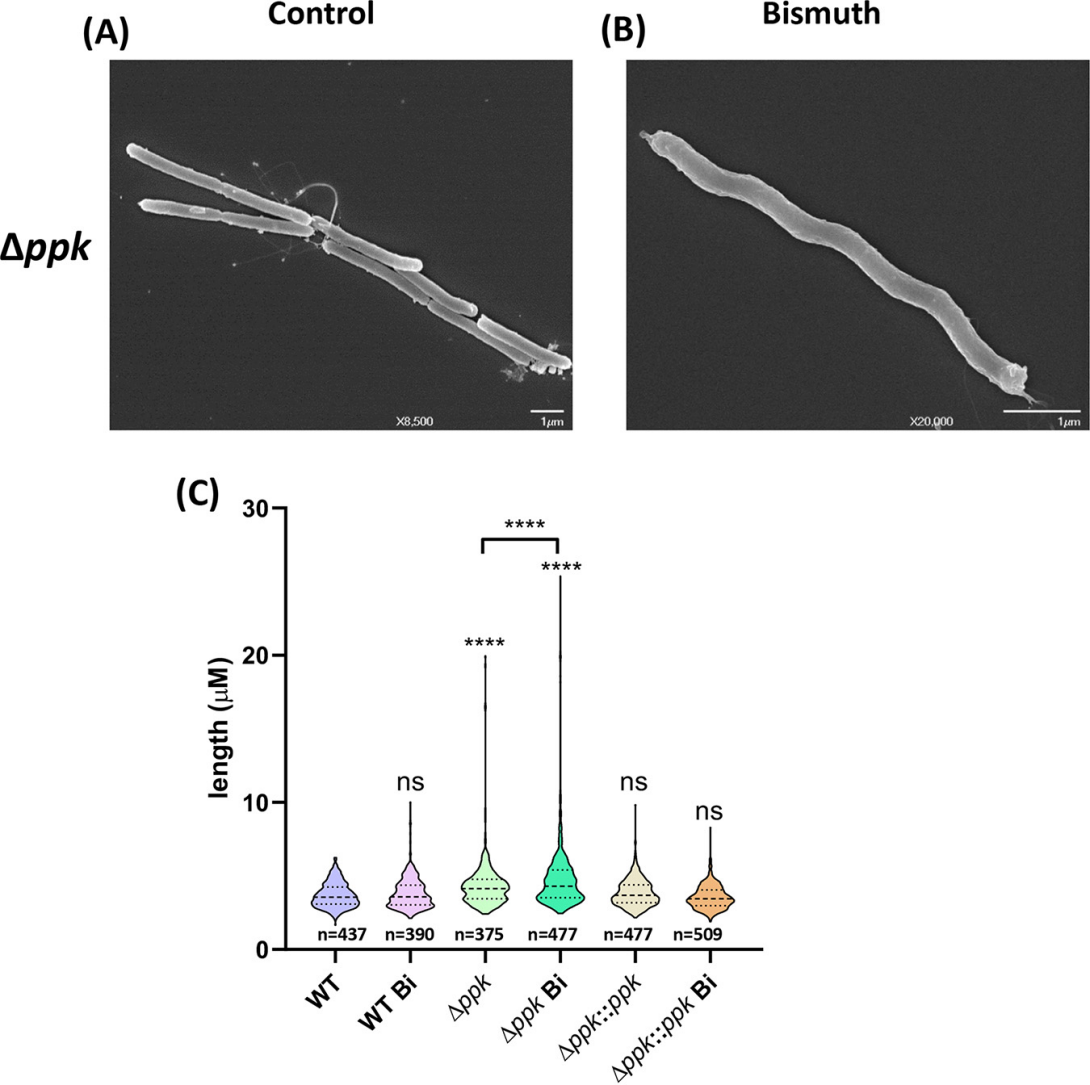
Ultrastructural analysis of the H. pylori Δ*ppk* mutant. (A and B) SEM of the H. pylori Δ*ppk* mutant under control conditions (A) or treated with bismuth (B). (C) Bacterial size distribution of wild-type, Δ*ppk* mutant, and complemented Δ*ppk::ppk* cells under control conditions or treated with bismuth. Images were taken by phase-contrast microscopy, and size measurements were performed with ImageJ software with the MicrobeJ plugin. Statistical analysis was carried out using a one-way ANOVA test (*n* > 400 for each condition). ****, *P* < 0.0001; ns, nonsignificant.

In addition, we observed that the H. pylori *Δppk* mutant presented an increased cell length (4.4 ± 1.8 μm) compared to that of the wild-type strain (3.7 ± 0.8 μm) (Fig. 4). Furthermore, the *Δppk* mutant cell length change was significantly accentuated upon bismuth exposure, resulting in frequent filamenting bacteria of >20 μm (Fig. 4). The average length of Δ*ppk* mutant cells upon bismuth exposure was significantly increased (4.9 ± 2.5 μm) that of the wild-type cells. We conclude that the formation of nonseparated daughter cells occurs in the *Δppk* mutant and that this defect is enhanced by bismuth. Wild-type cell segregation and separation were restored in the *Δppk* complemented strain (mean length, 3.8 ± 0.9 μm), with no significant modification by bismuth (3.6 ± 0.8 μm) (Fig. 4C). These results suggest that the absence of PPK activity prevents efficient cell segregation and that bismuth amplifies this phenotype.

### Bismuth-induced poly-P granules promote nucleoid reorganization.

**FIG 5:**
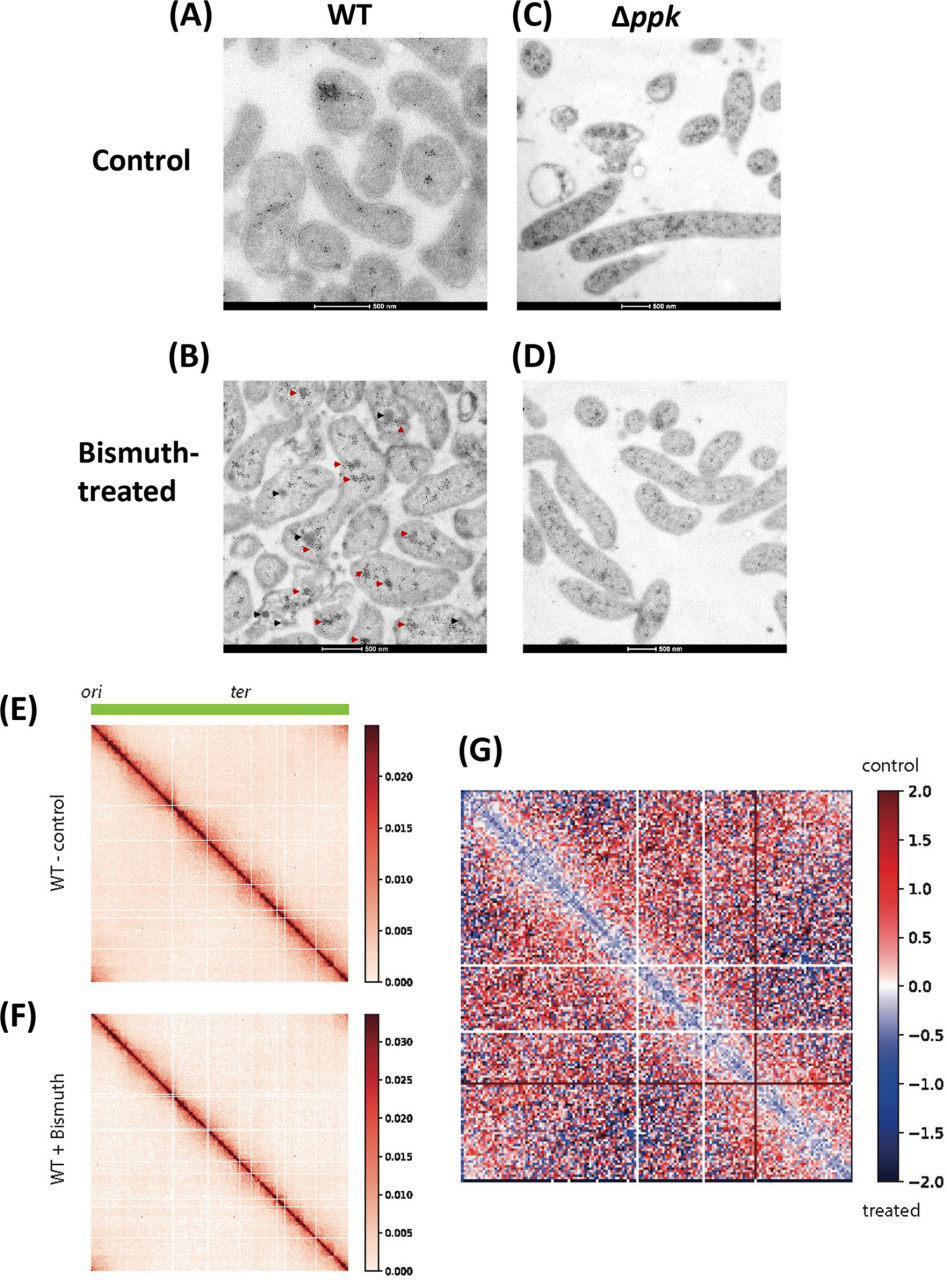
Bismuth exposure induces condensation of the H. pylori nucleoid. (A and B) TEM of H. pylori wild-type cells under control conditions (A) or treated with bismuth (B). (C and D) Sections of Δ*ppk* mutant cells under control conditions (C) or treated with bismuth (D). Wild-type cells, but not *Δppk* mutants, show condensation of the nucleoid structure upon bismuth treatment. Red arrowheads indicate the presence of DNA structures in the phosphorus-rich regions. Black arrowheads indicate the electron-dense structure not labeled with anti-DNA antibody. (E to G) Hi-C analysis of H. pylori chromosome structure of cells exposed or not to bismuth. (E and F) Normalized contact map of H. pylori exponentially growing cells not treated (E) or treated (F) with a sublethal concentration of bismuth. *x* and *y* axes indicate chromosomal coordinates binned in 5-kb bins (origin and terminus of replication are indicated at the top). (G) Ratio map (log_2_) of the two tested conditions (wild type not treated versus treated); color scales indicate contact enrichment in treated (blue) or not treated (red) cells (white indicates no difference between the two conditions).

Modifications in the nucleoid organization were previously observed in response to stress, which could have a global impact on gene expression (28). Therefore, we tested whether bismuth-induced poly-P granules could be associated with nucleoid reorganization in H. pylori. For this analysis, we first performed immunogold EM labeling with mouse monoclonal anti-DNA antibody. Cryosections from control bacteria exhibit numerous gold particles staining the DNA structures that are uniformly distributed throughout the bacterial cytoplasm (Fig. 5A). In accordance with the TEM data presented above (Fig. 2A and B), we observed electron-dense structures in cryosections of bismuth-exposed bacteria. Remarkably, gold particles frequently accumulated in electron-dense regions, suggesting local nucleoid compaction (Fig. 5B, red arrowheads). However, not all dense structures were stained with gold particles, suggesting either a different nature or a low antigenic availability of DNA within these structures (Fig. 5B, black arrowheads). Nevertheless, these results indicate that bismuth induces alterations of nucleoid organization. To precisely figure out the role of poly-P granules on nucleoid reorganization in response to bismuth, the Δ*ppk* mutant was examined. In contrast to the wild-type strain, no significant local DNA condensation was induced by bismuth in the absence of poly-P granules (Fig. 5C and D). In sum, these data suggest that bismuth induces alterations in nucleoid compaction that are mediated or influenced by the presence of poly-P granules. This is the first report showing that bismuth exposure combined with poly-P granules can alter nucleoid structures in H. pylori.

### Hi-C analysis validates the condensation of the H. pylori nucleoid upon bismuth treatment.

To see whether H. pylori chromosome architecture was affected by bismuth treatment, we applied the Hi-C approach to exponentially growing wild-type cells treated or not with bismuth (Fig. 5E to G). The contact map resulting from untreated cultures (5-kb bin) (Fig. 5E) displayed classical bacterial features, such as the broad and strong primary diagonal exhibiting small self-interacting regions visible as squares along this diagonal. On the other hand, unlike many model organisms studied so far (29–31), the H. pylori contact map did not exhibit an antidiagonal, suggesting that there is no chromosomal arm cohesion in this organism, just like in Escherichia coli (32). The contact map of the treated culture displayed the same overall architecture, showing that there is no major rearrangement of H. pylori chromosome folding after exposure to bismuth (Fig. 5F). However, some differences appeared between the two conditions, as shown by the resulting ratio map (10-kb bin) (Fig. 5G). Indeed, the bismuth treatment led to a clear decrease in long-range interactions, as indicated by the strong blue signal in the ratio map. Our results indicate that bismuth exposure induces clear alterations of nucleoid organization and especially its compaction.

## DISCUSSION

In Gram-negative bacteria, MV formation starts with outer membrane blebbing that ultimately ends with the release of MVs (33). In H. pylori, MVs are one of the crucial elements in disease development and persistent infections (34). H. pylori MVs have also been reported to play a protective role against exposure to oxidative stress and antimicrobial agents (35). A recent study has shown that the protein cargo of H. pylori MVs varies with the growth stage and that this cargo is dependent on the size of the MVs, a parameter that also determines their entry into host cells (36, 37).

In this study, we investigated the effect of a metal on the biogenesis and cargo content of H. pylori MVs. We found that when H. pylori is exposed to sublethal bismuth concentrations, the size distribution profile of secreted MVs is altered and the amount of membrane-bound MVs is increased potentially as a consequence of delayed MV shedding. One remarkable finding of our study is that MVs released from H. pylori bacteria exposed to bismuth accumulate bismuth and that no bismuth is detected in the bacterial cells. Differential proteomics analysis of the cargo content of bismuth-exposed MVs versus control MVs shows that bismuth accumulation is associated with selective enrichment in proteins from multiple biological pathways, in particular previously identified bismuth-binding proteins as well as iron- and nickel-binding proteins. These metal-binding proteins could be mediating the bismuth accumulation that we measured in MVs. We validated the bismuth induction of one such protein, SlyD, at the transcriptional and protein level and showed its enrichment in both bacteria and MVs purified from bismuth-exposed bacteria. SlyD has been shown to bind bismuth (21), and it could be one of the proteins which facilitates accumulation of bismuth in the MVs.

Among other metal-binding proteins enriched in bismuth-exposed MVs, UreE and HypB are well-characterized nickel accessory proteins involved in the maturation of urease and hydrogenase, respectively (38, 39). We previously characterized the abundant HspA, a GroES homolog, as a hydrogenase nickel metallochaperone and SlyD as a nickel-binding protein regulating nickel uptake by the Niu transporter (14, 22). One of the few H. pylori global regulators, the iron-responsive Fur protein is more abundant in MVs of bismuth-treated bacteria. Fur is crucial in maintaining iron uptake, iron storage, and urease expression in H. pylori (40–42), and binding of bismuth inactivates its function (43). Transcriptional deregulation caused by Fur inactivation could be one mechanism of differential expression in the presence of bismuth. In addition, the CadA, HbpA, and FrpB2 proteins, which are less abundant in MVs of bismuth-treated bacteria, are particularly interesting, since their downregulation is a signature of their probable role in metal and possibly bismuth uptake.

Together, these data show that bismuth, even at low doses, penetrates the cells and, most probably by interfering with Fur and other metal-binding and metabolic proteins, results in a drastic change in MV protein cargo content. The observed intracellular poly-P granule accumulation induced by bismuth might also globally impact gene expression in H. pylori, as it was reported that poly-P interacts with the housekeeping sigma factor (σ^80^) in H. pylori and that the absence of this interaction during starvation stress leads to cell death (44). We cannot exclude that NikR, a nickel-responsible regulator, is also playing a role in this response.

Our data suggest that MVs are utilized by H. pylori as a vehicle to selectively discard bismuth out of the cell and thereby escape its toxicity. In addition, bismuth also acts at the level of MV production by slightly affecting the size distribution of secreted MVs and thereby possibly regulates the well-documented interaction of MVs with host cells. Although previously hypothesized (45), a specific role of bacterial MVs in metal expulsion from bacteria has recently been suggested for copper in the cyanobacterium *Synechocystis* sp. strain PCC 6803. However, in that study, only a mutant of the TolC outer membrane protein presented hypervesiculation in response to high copper doses and a copper chaperone (CopM) was detected in MVs in association with enhanced copper (46).

In other bacterial species, MVs have been reported to carry iron and zinc acquisition systems that are proposed to play a role in providing bacteria with access to these essential compounds (47). In Leptospira interrogans, a mutant deficient in HtpX, an outer membrane metalloprotease, releases MVs and upon a challenge with toxic iron concentrations displays passive iron precipitation at the bacterial surface (48). These data also contrast with our findings, since accumulation of metal was observed only in the L. interrogans mutants, probably as a consequence of intense envelope stress, and was not specific to MVs.

Previous studies have shown that exposure of H. pylori to toxic amounts of bismuth (up to 1 mg mL^−1^ CBS, 1,000 times the concentration used here), besides causing cell lysis and changing the wall structures, induces the appearance of electron-dense cytoplasmic aggregates with a vacuole-like center that was assigned to intracellular accumulation of bismuth (18, 19). Under our conditions, no cell lysis was observed but very similar electron-dense structures were visualized. We were able to demonstrate that these structures are not composed of bismuth but rather are rich in phosphorus. More precisely, these structures were identified to be poly-P granules resulting from the activity of PPK. In addition, several sections had “white vacuole-like” structures which were found to have a phosphorus-rich periphery. We postulate that these vacuole-like structures correspond to brittle poly-P crystalline structures that are discarded during the 60-nm-section cutting steps of TEM sample preparation that includes prior extensive dehydration. In favor of this explanation is the observation that (i) the H. pylori *Δppk* mutant failed to display these granules and vacuole-like structures in response to sublethal bismuth and (ii) these vacuole-like structures are not observed during the Tokuyasu sample preparation for TEM immunogold analysis, a procedure that does not include a strong dehydration step.

The PPK protein was previously characterized in H. pylori, and its function was shown to be essential for mouse colonization (25). Poly-P formation is an ancient and ubiquitous starvation response serving diverse functions in both prokaryotes and eukaryotes, ranging from as a reservoir of phosphorus and energy, in nutrient stress adaptation, or as a signaling molecule (49). Poly-P granules have been involved in the acquisition of heavy metal resistance in archaea, bacteria, and fungi (50). However, the only demonstrated mechanism implies that the metal is associated with poly-P before the latter is degraded by the action of exopolyphosphatase (PPX) and further extruded under the form of metal-HPO_4_^−^ (51, 52). This is different from the response of H. pylori reported here. In our study, accumulation of poly-P granules in response to bismuth might be mediated by a slight induction of PPK protein. We also observed that formation of poly-P granules in response to bismuth was required neither for the bismuth accumulation in the MVs nor for the resistance of H. pylori to toxic bismuth concentrations. Therefore, while we can conclude that poly-P granule formation is a response induced by bismuth, more work is needed to define the underlying regulatory mechanisms and explore a possible function in metal protection or detoxification.

We also revealed another role of poly-P granules in H. pylori, linking these structures to cell division and nucleoid rearrangement. A significant increase in cell length was seen in the *Δppk* mutant, as previously reported (26). This phenotype was further amplified upon bismuth exposure, leading to filamentation of H. pylori. We also observed that poly-P granules are spatially associated with the H. pylori nucleoid and that bismuth induces local condensation of nucleoid structures. A first Hi-C analysis was performed for H. pylori, revealing that there was no chromosomal arm cohesion in this organism. Importantly, Hi-C analysis of H. pylori cells exposed to bismuth evidenced compaction of the chromosome under this condition.

The *Δppk* mutants do not present such nucleoid condensation upon exposure to bismuth. We propose that poly-P granules would locally protect the nucleoid from bismuth stress. In the absence of poly-P granules, bismuth-mediated stress would not be well tolerated and would lead to alterations in nucleoid dynamics and inefficient partitioning of the chromosome in the daughter cell that results in a default in their separation upon division.

In Caulobacter crescentus, a close association of the chromosome and poly-P granules has been observed in which segregation of chromosomes governs poly-P biogenesis and distribution to the daughter cell (53). Physical association of poly-P with bacterial nucleoid structures was earlier seen in different species (54–56), further implying a crucial dynamic role of poly-P in nucleoid assembly across different species.

Bismuth-mediated induction of poly-P granules and the role of poly-P in maintaining an efficient separation of daughter cells and thus cell length highlight the crucial need to further understand poly-P functions in H. pylori. Although we discovered poly-P and nucleoid association upon bismuth exposure, we do not yet know whether or not this association is mediated by protein factors. Since DNA and poly-P are both negatively charged in nature, we cannot rule out the possibility of an additional third factor in the assembly. The only protein of the Dps family of H. pylori, the neutrophil activating protein NapA, is known to bind both iron and DNA. NapA can also bind bismuth *in vitro* (21). A possible hypothesis is that upon bismuth binding and in association with poly-P, the activity of the NapA protein is modified, resulting in nucleoid compaction. Unfortunately, this could not be tested, because we found that *napA* is an essential gene in H. pylori strain G27. The molecular benefit of poly-P and nucleoid interactions could have physiological roles that need to be studied in detail to better understand the response of H. pylori to metal toxicity.

**FIG 6:**
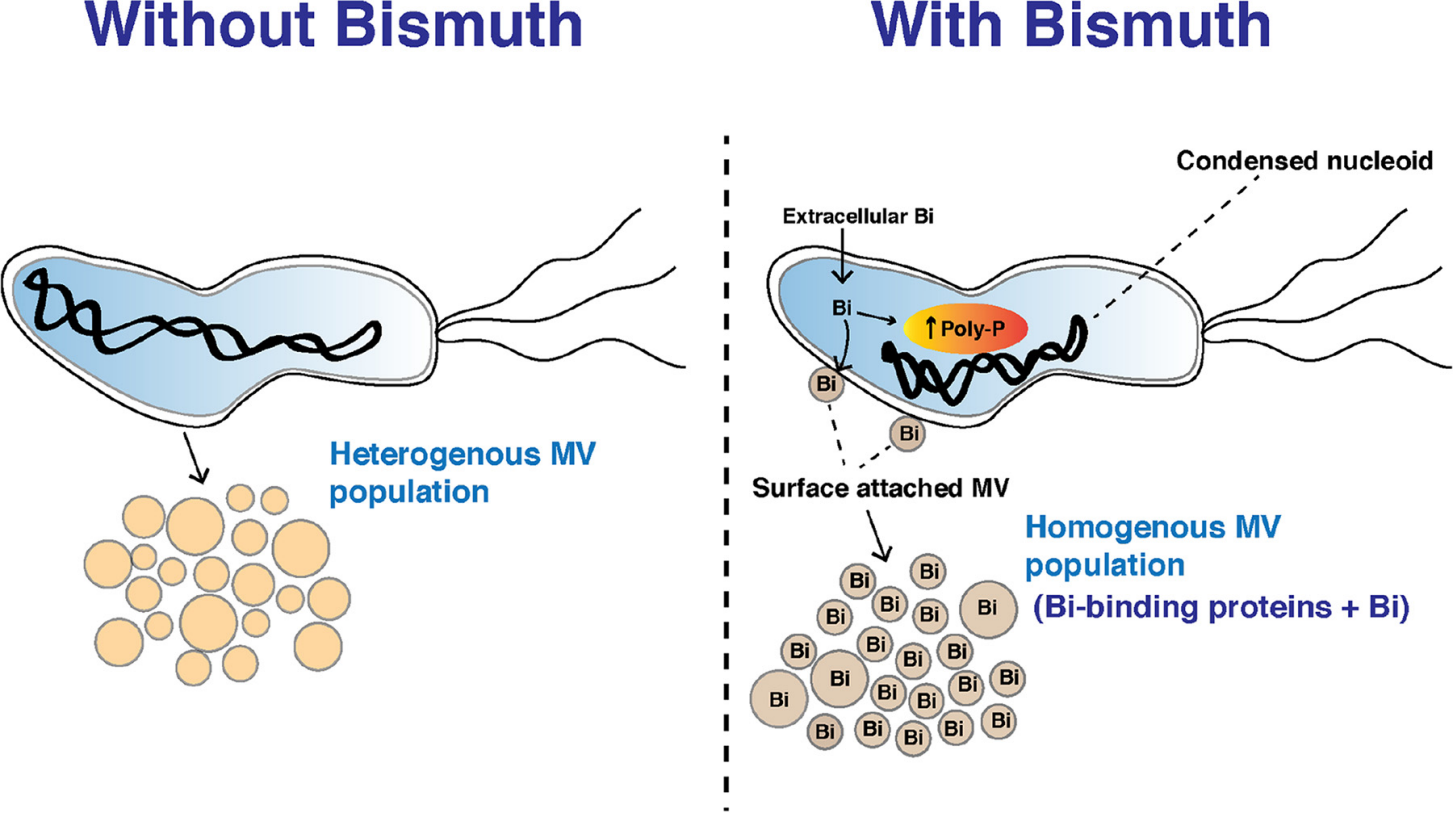
Schematic representation of the consequences of H. pylori exposure to sublethal bismuth concentrations. Bismuth is taken up by H. pylori cells, induces the formation of poly-P granules in the cytoplasm, and causes nucleoid condensation. Bismuth induces the formation of homogenous membrane vesicles that are enriched in bismuth-binding proteins and in the metal bismuth. We conclude that H. pylori eliminates the toxic bismuth by packaging this metal together with bismuth-binding proteins in membrane vesicles and locally protects its chromosome from metal toxicity.

To summarize our findings, we observed multilevel effects of a sublethal exposure of bismuth on H. pylori physiology, implying protection and defense mechanisms (summarized in a model shown in Fig. 6). Since blood plasma concentrations of bismuth (an accepted proxy for gastric concentration) are in the sublethal range for H. pylori, our study provides a first framework analysis of the effects of and the bacterial protection against bismuth exposure. We discovered a novel defense mechanism employed by H. pylori to escape bismuth toxicity by shedding bismuth into the environment via MVs (Fig. 6).

We propose that the quadruple bismuth therapy used against H. pylori could be further improved by adding inhibitors of bacterial MV formation, which should reduce the capacity of H. pylori to expel bismuth. Since MVs are produced by both Gram-negative and -positive bacteria, our study lays out an initial foundation to better understand the role of MVs in metal trafficking. The second highlight of our study focuses on the interplay of poly-P granules and the organization of the nucleoid structure in response to bismuth. We propose that accumulation of poly-P and its spatial interaction dynamics could be a local shielding mechanism employed by bacteria against bismuth toxicity.

## MATERIALS AND METHODS

### Bacterial strains and growth conditions.

H. pylori strain G27 (20) and derivatives are listed in Table S3A in the supplemental material. H. pylori strains were grown on blood agar base 2 (Oxoid) plates supplemented with 10% defibrinated horse blood (Invitrogen) and an antibiotic-antifungal cocktail (vancomycin, 12.5 μg mL^−1^; trimethoprim, 6.25 μg mL^−1^; polymyxin B, 0.31 μg mL^−1^; and amphotericin B 2.5 μg mL^−1^) under microaerobic conditions. For liquid cultures, bacteria were grown in Brucella broth (BD Difco) supplemented with 10% fetal calf serum (FCS; Eurobio) and an antibiotic-antifungal cocktail under constant shaking at 150 rpm. H. pylori was grown at 37°C under microaerophilic conditions (6% O_2_, 10% CO_2_, 84% N_2_) generated with an Anoxomat (MART Microbiology) atmosphere generator.

10.1128/mbio.01633-22.10 TABLE S3 (A) List of strains used in the study. (B) List of primers used in the study. Download Table S3, DOCX file, 0.02 MB. Copyright © 2022 Kumar et al. 2022 Kumar et al. https://creativecommons.org/licenses/by/4.0/ This content is distributed under the terms of the Creative Commons Attribution 4.0 International license.

### Bismuth MIC determination.

Bismuth(III) citrate (Sigma) could be efficiently dissolved in 10% liquid ammonia (pH adjusted to 10.5) at a concentration of 500 mg mL^−1^, yielding a 1.2 M soluble stock solution. To test if the dilution of bismuth to neutral pH medium will result in formation of precipitate, control experiments were done in which different concentrations of bismuth were added to the culture medium and the medium was followed for 24 h for the occurrence of precipitate. No visible precipitate was seen in the medium upon bismuth addition, and centrifugation of the culture medium did not result in precipitate formation. The MIC of bismuth was determined by the agar dilution method by plating serial dilutions of 2 × 10^7^ H. pylori CFU on Brucella agar plates that contained bismuth in a 2-fold dilution series ranging from 321 μM to 2.50 μM. CFU were counted after 6 days of incubation under microaerophilic conditions. The MIC was defined as the minimum concentration of bismuth needed to completely inhibit the growth of H. pylori on an agar plate. For a control, we verified that the MIC for bacteria exposed to the vehicle solution in which bismuth was prepared (but without adding bismuth) was the same as the MIC for nonexposed bacteria.

### Molecular techniques.

Molecular biology experiments were performed according to standard procedures and supplier (Fermentas) recommendations. A NucleoBond Xtra Midi kit (Macherey-Nagel) and a QIAamp DNA minikit (Qiagen) were used for plasmid preparations and H. pylori genomic DNA extractions, respectively. PCR was performed with either DreamTaq DNA polymerase (Thermo Fisher) or Q5 DNA polymerase (NEB).

### Construction of H. pylori mutant strains.

The open reading frame (ORF) of the *ppk* gene encoding polyphosphate kinase in H. pylori strain G27 (*hpg27_*418) is 2,028 bp long. We constructed a mutant carrying a large internal deletion from bp 421 to 1620 by allelic replacement with a nonpolar kanamycin resistance (Kan) cassette as described in reference 15 (Table S3A). The Kan cassette (819 bp) was PCR amplified using primers shown in Table S3B. A 400-bp-long region upstream of position 421 of the *ppk* gene was PCR amplified, and a 20-bp overlap was introduced with the 5′ region of the Kan cassette. Similarly, a 400-bp-long region downstream of position 1620 was PCR amplified, and a 20-bp overlap was introduced with the 3′ region of the Kan cassette. Fusion PCR was carried out to fuse the 400-bp upstream and downstream regions with the Kan cassette using a two-step PCR. The corresponding PCR product of 1,619 bp was purified and used for natural transformation of strain G27. Mutants were selected on blood agar plates containing 20 μg mL^−1^ of kanamycin. Genomic DNA of single colonies was used to validate the *ppk* deletion in the mutant using primers indicated in Table S3B.

On-locus complementation of the *ppk* gene was carried out using an apramycin resistance cassette as a selection marker in the G27 Δ*ppk* mutant strain. First, the ORF of the *ppk* gene along with a 500-bp upstream region was PCR amplified to add a 27-bp FLAG tag coding sequence at the 3′ end of the *ppk* ORF region (primers in Table S3B). The apramycin cassette (805 bp) was PCR amplified to introduce a 27-bp overlap with the FLAG tag coding sequence at the 5′ end. Similarly, a 500-bp downstream region of the *ppk* ORF was PCR amplified to add a 28-bp overlap at the 5′ end with the 3′-end region of an apramycin resistance gene. All three fragments were fused by a two-step PCR to form a 3,857-bp linear product. A PCR-purified fragment of 3,857 bp was then used to naturally transform the G27 Δ*ppk* mutant strain, and recombinants were selected on a blood agar plate containing 10 μg mL^−1^ apramycin. Genomic DNA isolated from single colonies was used to screen genetic locus-complemented strains of *ppk*. Insertions were verified by PCR amplification.

### RNA extraction and cDNA synthesis.

The total RNA of bismuth-treated and control samples was isolated from 1 mL of H. pylori culture with an optical density at 600 nm (OD_600_) of 1.0 corresponding to 10^8^ CFU. Bismuth-treated samples were grown in the presence of 2.5 μM bismuth for 6 h. Quantitative real-time PCR was performed as described by Denic et al. (22). The transcript levels were normalized to the level of the 16S rRNA. The data correspond to at least two independent experiments with two technical replicates each time.

### Purification and characterization of H. pylori membrane vesicles.

Exponentially growing cultures of H. pylori were diluted to an OD_600_ of 0.15 in 200 mL Brucella broth medium supplemented with 10% fetal calf serum (FCS) and antibiotic cocktail. For bismuth treatment, a sublethal dose of bismuth was added at a final concentration of 2.5 μM in the medium. Bacterial culture was grown with constant shaking under microaerobic conditions until an OD_600_ of 0.50 was reached. To isolate secreted MVs, present in the growth medium, bacterial mass was first harvested by centrifugation at 12,000 × *g* for 20 min, followed by filtration of the supernatant through a 0.22-μm polyethersulfone (PES) membrane. The filtered supernatant (500 mL) was concentrated to 22 mL by using a 100-kDa Centricon tube. Concentrated supernatant was subjected to ultracentrifugation at 100,000 × *g* for 180 min to pellet the MVs. The MV pellet was resuspended in 200 μL of phosphate-buffered saline (PBS) and washed at least twice by a 30-min spin at 100,000 × *g* before final suspension in 200 μL of PBS.

For ICP-OES analysis, MVs were further purified by density gradient centrifugation using OptiPrep solution (Sigma). A discontinuous OptiPrep density gradient was prepared in a 5-mL volume consisting of 50%, 30%, and 10% Optiprep solution, and MVs resuspended in PBS were layered at the top of the gradient. Centrifugation was carried out at 100,000 × *g* for 16 h at 4°C. A band of MVs was collected using a 1-mL needle and washed twice with PBS solution for 1 h at 100,000 × *g* and 4°C. The resulting pellet was finally vacuum dried and processed for ICP-OES analysis.

Purified MVs were stored at −20°C. The quality of the preparation and the absence of contaminating bacteria were validated by TEM (Fig. S1D and E). The MV purification yields (particles/mL culture) were measured by NanoSight tracking analysis and were similar for the two conditions, i.e., with and without bismuth.

### DLS of purified MVs.

Dynamic light scattering (DLS) analysis was performed on purified MVs using a DynaPro plate reader (Wyatt, Santa Barbara, CA). Data were recorded at 20°C with 20 acquisitions, each lasting 10 s. Three sets of data were acquired for each sample with two independent biological replicates. Particle size determination was done using Dynamics V7 software (Wyatt). The intensity-weighed distribution of vesicles was plotted against diameter size. DLS measurements gave diameter data of the vesicles, which comprise water of hydration, and thus provided subaccurate measurements of MV diameters. The resulting variation seen in the sizes of MVs by DLS and TEM measurements was due to the technical limitation of the DLS technique.

### Comparative proteomics analysis of purified MVs.

**(i) Sample preparation.** For comparative proteomics analysis, MVs were purified from 150 mL of bacterial culture grown until an OD_600_ of 0.5 was reached in the presence and absence of 2.5 μM bismuth. Purification of MVs was done as mentioned above, except that the bacterial MVs were resuspended in 150 μL of PBS and washed four times in PBS to remove any medium component contamination. Final resuspension was performed in 150 μL of PBS containing 1× cocktail protease inhibitor, 5 mM 2-mercaptoethanol, and 2% SDS. Sonication was carried out to lyse the MVs and was followed by centrifugation at 14,000 × *g* to eliminate debris. Proteins were quantified, and 200 μg of proteins for each sample was briefly electrophoresed on a precast 10% stain-free SDS gel (Bio-Rad). Sample bands were visualized using InstantBlue Coomassie protein stain (Abcam), and bands were excised for in-gel trypsin digestion.

**(ii) Protein digestion.** In-gel tryptic digestion was performed as described previously (57). Briefly, gel slices were destained in 100 mM ammonium bicarbonate-acetonitrile (1:1). Samples were reduced (10 mM dithiothreitol [DTT] in 100 mM ammonium bicarbonate, 30 min at 56°C) and alkylated (55 mM iodoacetamide in 100 mM ammonium bicarbonate, 30 min at room temperature in the dark). Proteins were digested using 2 μg of sequencing-grade modified trypsin (Promega, Madison, WI, USA) in 10 mM ammonium bicarbonate overnight at 37°C. The resulting peptides were extracted with formic acid to a 5% final concentration and desalted on a StageTip (58) prepared with 3M Empore C_18_ material (Fisher Scientific).

**(iii) LC-MS analysis.** Tryptic peptides were analyzed on a Q Exactive Plus mass spectrometer (MS) (Thermo Fisher Scientific, Bremen, Germany) coupled with an EASY-nLC 1200 chromatography system (Thermo Fisher Scientific, Bremen, Germany). Peptide digests (1 μg) were loaded and separated at 250 nL min^−1^ on an in-house-packed 30-cm nano-high-performance liquid chromatography (HPLC) column (75-μm inner diameter) with C_18_ resin (1.9-μm particles, 100-Å pore size, Reprosil-Pur Basic C_18_-HD resin; Dr. Maisch GmbH, Ammerbuch-Entringen, Germany) equilibrated in 97% solvent A (H_2_O, 0.1% FA) and 3% solvent B (acetonitrile (ACN), 0.1% formaldehyde (FA)). Peptides were eluted with a linear gradient of 3% to 29% buffer B for 70 min, followed by a stepwise increase of buffer B to 56% B for 30 min, and finally to 100% for 5 min.

Mass spectra were acquired with a Top10 data-dependent acquisition mode, with the scan range set to *m/z* 300 to 1,700 and an automatic gain control (AGC) target value of 3 × 10^6^. The fragmentation of precursor ions was performed by high-energy collisional dissociation (HCD) (normalized collision energy [NCE], 28) at 17,500 resolving power (at *m/z* 200) with an AGC target value of 1 × 10^6^ and a maximum injection time of 60 ms. Precursors with an unknown charge state or a charge state of 1 and >7 were excluded. Dynamic exclusion was set to 45 s.

**(iv) MS protein identification and quantification.** All data were searched using MaxQuant version 1.5.3.8 against the Helicobacter pylori G27 UniProt reference proteome database (1,501 entries) concatenated with the usual known MS contaminants and reversed sequences of all entries. Andromeda searches were performed with trypsin chosen as a specific enzyme with a maximum number of 4 missed cleavages. Possible modifications included carbamidomethylation (Cys, fixed), oxidation (Met, variable), and N-terminal acetylation (variable). The mass tolerance in MS was set to 20 ppm for the first search and then 6 ppm for the main search and 10 ppm for the tandem MS (MS/MS). The maximum peptide charge was set to 7, and five amino acids were required as the minimum peptide length. The maximum peptide mass was increase to 8,000 Da. The “match between runs” feature was applied between replicates with a maximal retention time window of 0.7 min. One peptide unique to the protein group was required for the protein identification. A false discovery rate (FDR) cutoff of 1% was applied at the peptide and protein levels. The MaxLFQ, i.e., Maxquant’s label-free quantification, algorithm was used to calculate protein intensity profiles across samples. The iBAQ feature was selected for further quantitative analysis (see Data Set S1 in the supplemental material). The MS proteomics data have been deposited in the ProteomeXchange Consortium via the PRIDE partner repository under the data set identifier PXD028509.

10.1128/mbio.01633-22.6 DATA SET S1 Pairwise quantitative analysis of proteins identified in MVs purified from bismuth-exposed bacteria versus control conditions. Download Data Set S1, XLSX file, 0.4 MB. Copyright © 2022 Kumar et al. 2022 Kumar et al. https://creativecommons.org/licenses/by/4.0/ This content is distributed under the terms of the Creative Commons Attribution 4.0 International license.

### Statistical analysis of proteomics data.

For the differential analyses, proteins identified in the reverse and contaminant databases and proteins “only identified by site” were first discarded from the list of identified proteins. Then, proteins exhibiting fewer than two quantified values in at least one condition were discarded from the list. After log_2_ transformation of the protein intensity values, these values were normalized by median centering within conditions (wrapper.normalizeD function of the R package DAPAR [59]). The remaining proteins without any intensity value in one of the two conditions have been considered proteins quantitatively present in a condition and absent in another. They have therefore been set aside and considered differentially abundant proteins. Next, missing values were imputed using the impute.mle function of the R package imp4p (60). Proteins with a fold change of less than 2 have been considered not significantly differentially abundant. Statistical testing of the remaining proteins (with a fold change greater than 2) was conducted using a limma *t* test from the R package limma. An adaptive Benjamini-Hochberg procedure was applied to the resulting *P* values using the function adjust.p of R package cp4p (61). The proteins associated with an adjusted *P* value inferior to an FDR threshold of 1% have been considered significantly differentially abundant proteins. The proteins of interest are therefore those which emerge from this statistical analysis supplemented by those which are considered to be absent from one condition and present in another (Data Set S1).

### Functional analysis of the proteins of interest identified by proteomics.

The identified proteins of H. pylori strain G27 were mapped to proteins of other strains (strain ATCC 700392, 26695, or P12) if they shared at least 90% of the same amino acid sequences, in order to infer additional annotations (GO terms and KEGG pathways) (Data Sets S1 and S2). From these annotations, an overrepresentation analysis was performed using hypergeometric tests to assess the overrepresentation hypothesis for each GO term/KEGG pathway using R software. All the proteins identified by MS were used as background for the hypergeometric tests. A significantly low enrichment *P* value means that the proportion of proteins related to a GO-term/KEGG pathway is significantly superior in the considered list than in this background.

The protein-protein interaction network of the proteins found to be significantly more abundant in “bismuth” samples than in “control” samples was determined from STRING v11 (62) and visualized using Cytoscape v3.8.0 (63) with the plugins (Cytoscape) stringApp and Omics Visualizer (64). The widths of the edges correspond to the combined score from STRING, reflecting the confidence placed in each interaction. The different categories of proteins were determined from enriched KEGG pathways (enrichment *P* < 10%). We also highlighted the categories of interest of “bismuth-binding,” “metal ion-binding,” and “zinc ion-binding” proteins, using GO terms and literature (Data Set S2).

10.1128/mbio.01633-22.7 DATA SET S2 Functional analysis of the proteins of interest identified by proteomics. Download Data Set S2, XLSX file, 0.03 MB. Copyright © 2022 Kumar et al. 2022 Kumar et al. https://creativecommons.org/licenses/by/4.0/ This content is distributed under the terms of the Creative Commons Attribution 4.0 International license.

### Western blot analysis of bacterial lysate and MVs.

Exponentially growing bacterial samples were harvested and resuspended in the lysis buffer (25 mM Tris-Cl [pH 7.4], 150 mM NaCl, 5 mM 2-mercaptoethanol, and 1× protease inhibitor). Cell lysis was done by sonication, followed by centrifugation to remove cellular debris at 12,000 × *g* for 20 min. Following centrifugation, supernatant was collected and used for Western blot analysis of the SlyD and PPK-FLAG proteins. Purified MVs were fractionated to separate the soluble fraction from the membrane components by use of a previously established procedure (22). First, purified MVs were resuspended in the lysis buffer (25 mM Tris-Cl [pH 7.4], 150 mM NaCl, 5 mM 2-mercaptoethanol, 0.1% NP-40, and 1× protease inhibitor). Lysis was carried out by sonication, and lysed samples were briefly centrifuged at 12,000 × *g* before they were subjected to ultracentrifugation at 100,000 × *g* for 1 h. Supernatant after ultracentrifugation was collected and used for Western blot analysis of SlyD protein.

For Western blots, 50 μg of cell lysates or 200 μg of MV soluble fractions for SlyD detection was loaded and separated on any-kD Mini-Protean TGX stain-free precast protein gel (Bio-Rad) and subsequently transferred to a polyvinylidene difluoride (PVDF) membrane (Bio-Rad) with the TransBlot Turbo system (Bio-Rad). The H. pylori SlyD and PPK-FLAG proteins were detected with rabbit polyclonal anti-SlyD antibodies (22) and with monoclonal anti-FLAG M2 antibodies produced in mice (F3165, Sigma) at 1:3,000 and 1:1,000 dilutions, respectively. Secondary rabbit anti-mouse IgG (Promega) for anti-FLAG or goat anti-rabbit IgG antibody (Promega) conjugated with horseradish peroxidase (HRP) was added at a 1:40,000 dilution. Detection was achieved with ECL Plus reagent (Thermo Fisher). Images were taken with a ChemiDoc MP imaging system (Bio-Rad).

### Measurement of intracellular bismuth content in H. pylori cells and in MVs using ICP-OES.

Liquid cultures of exponentially growing H. pylori strain G27 were diluted and used to inoculate fresh culture at a starting OD_600_ of 0.10 in the presence and absence of 2.5 μM bismuth. Bacteria were further grown until the OD_600_ reached 0.5 (approximately 6 h of growth). Bacteria were harvested by centrifugation of culture at 12,000 × *g* for 20 min. Bacteria were washed with PBS before the samples were stored at −20°C for further processing. For the preparation of MVs, culture supernatant was filtered through a 0.22-μm PES membrane and MVs were purified as described above. MVs were washed twice with PBS. Washed MVs were dried using a SpeedVac at 60°C for 2 h.

Bacterial mass was resuspended in 500 μL of cold PBS containing 1 mM EDTA, followed by mixing with 400 μL of a 1:2 mixture of the silicone oils AR20 and AR200 (Wacker) in accordance with a protocol that we previously validated (22). Samples were harvested at 8,000 × *g* for 10 min, and supernatant was removed. The bacterial pellet was dried in a SpeedVac at 60°C for 2 h. Final pellets of MVs and bacteria were mineralized overnight with a solution mix of 500 μL of 69% nitric acid (EMSURE) and 500 μL of 96% sulfuric acid (Alfa Aesar). After the mineralization step, Milli-Q water was added in each sample to a final volume of 20 mL. The bismuth content was measured by ICP-OES with an Agilent 720 series with axially viewed plasma and with a Bi calibration curve of 10 to 1,000 ppb at the Institut Lavoisier de Versailles. The content of Bi was determined using a standard curve prepared by calibrating the machine with certified ICP-grade Bi standards. The amount of bismuth was determined by measuring the emission wavelength at three wavelengths (222.828, 223.061, and 306.771 nm). The measurement of each strain under each condition was performed in at least three biological duplicates. Data were measured in two technical replicates for each sample. The results are presented as the percentage of the ratio of bismuth mass versus total sample mass used for the analysis.

### TEM analysis of purified membrane vesicles.

Purified MVs (10 μL) were allowed to adhere to alcian blue-treated (1%) (Sigma-Aldrich) 300-mesh Formvar-Cu-coated grids (Electron Microscopy Science) for 15 min. Samples were then fixed on the grids with 2.5% glutaraldehyde in 0.1 M HEPES buffer, pH 7.2, followed by staining with 2% aqueous uranyl acetate (for 30 s). Grids were washed three times with ultrapure water and air-dried. Dried grids were imaged with a Tecnai Spirit 120-kV TEM machine equipped with a bottom-mounted Eagle 4kx4k camera (FEI-Thermo Fisher). TEM images of MVs were taken at a magnification of ×18,500. For size distribution analysis, a total of 600 MVs for each condition were analyzed on TEM images using ImageJ software, and the diameter size was measured as per the scale bar of the image. Diameter data of MVs were size distributed with a window bin size of 10 nm to create a histogram plot. Nonlinear curve fitting using a Gaussian equation {*Y* = amplitude · exp(−0.5) ·[(*X* − mean)/SD]^2^} (where SD is the standard deviation) on the histogram data was done to obtain the frequency distribution plot.

### Bacterial sample preparation for SEM analysis.

Liquid cultures of H. pylori G27 strain grown overnight were inoculated in 10 mL of liquid medium at an OD_600_ of 0.15 with and without bismuth (2.5 μM). After 6 h of growth, bacterial cultures at an OD_600_ of 0.5 were fixed in solution by being mixed with a 10-mL solution of 5% glutaraldehyde for 15 min at room temperature, followed by overnight fixing at 4°C. Fixed bacteria were then harvested and washed in 0.1 M HEPES buffer (pH 7.2) and postfixed for 1 h 30 min in 1% osmium tetroxide in 0.1 M HEPES buffer (pH 7.2), followed by rinsing with distilled water. Samples were dehydrated through a graded series of 25, 50, 75, 95, and 100% ethanol solution, followed by critical point drying with CO_2_. Dried specimens were sputtered with 20-nm gold palladium with an ion beam coater and examined and photographed with a JEOL JSM 6700F field emission SEM operating at 7 kV. Images were acquired with the upper secondary electron (SE) detector in-lens upper secondary electron detector (SEI).

### Bacterial sample preparation for TEM analysis.

TEM analysis was performed on the fixed bacterial culture as prepared for SEM analysis. After fixation with glutaraldehyde and postfixation with osmium tetroxide, samples were resuspended in a 4% solution of agar type 9 and allowed to solidify on ice, dehydrated in a graded series of ethanol, and embedded in EMbed 812 epoxy resin. After heat polymerization, thin sections were cut with a Leica Ultracut UC7 ultramicrotome, and sections (60 nm) were stained with and without uranyl acetate and lead citrate. Images were taken with a Tecnai Spirit (FEI-Thermo Fisher Company) at 120 kV accelerating voltage with an Eagle 4kx4k camera (FEI-Thermo Fisher Company).

### Scanning-transmission electron microscopy (STEM) analysis of bacterial sections.

Bacterial samples were processed as described above for TEM analysis, and 60-nm sections were cut with a Leica Ultracut UC7 ultramicrotome. Sections were stained with uranyl acetate, and stained sections were visualized on carbon film (300 mesh). TEM measurements were performed using a JEOL (Japan) 2100F field emission gun instrument operating at 200 kV. Images were recorded on a Gatan UltraScan 4000 (USA) camera with a 4096 × 4096 charge-coupled device (CCD). To obtain the elemental composition of the bacterial sections, a JEOL X-ray energy dispersive spectra (XEDS) detector with 140-eV resolution was used. XEDS characterizing the elemental composition of the sample were recorded for both control and bismuth-treated samples. STEM analysis was carried out by using a TEM coupled-scanning TEM JEOL detector with a 1-nm probe operating in a high-angle annular dark-field (HAADF) mode. A combination of XEDS and STEM data revealed elemental composition mapping of the bacterial sections.

### Sample preparation and immunolabeling by the Tokuyasu technique for TEM.

Bismuth-treated (2.5 μM) and untreated cultures of H. pylori G27 wild-type strain and the Δ*ppk* mutant were fixed by mixing them with an equal volume of 2× fixative (0.2% glutaraldehyde [Sigma]) and 8% paraformaldehyde (EMS) for 15 min at room temperature, followed by overnight incubation at 4°C. Fixed samples were harvested and resuspended in PBS with 10% gelatin (Sigma). The samples were allowed to solidify on ice and infiltrated overnight with 2.3 M sucrose at 4°C. Samples were mounted onto sample pins and frozen in liquid nitrogen. Subsequently, samples were cryosectioned (60 nm thickness) using an FC6/UC6 cryoultramicrotome (Leica) and a 35° diamond knife (Diatome). The sections were picked up using a 1:1 mixture of 2% methylcellulose (Sigma-Aldrich) and 2.3 M sucrose. Samples were quenched with 50 mM NH_4_Cl, blocked in PBS with 1% bovine serum albumin (BSA) and immunolabeled with anti-DNA monoclonal antibody (Progen AC-30-10) diluted 1:5 for wild-type samples treated and untreated with bismuth and 1:20 for the Δ*ppk* mutant, in 1% BSA prepared in PBS. Staining with anti-DNA antibody was followed by a bridge step labeling with a polyclonal rabbit anti-mouse immunoglobulin (Dako Z0259: dilution = 1/50) and then final staining with protein A-gold (10 nm) for 15 min. Samples were fixed with 2.5% glutaraldehyde in 1× sodium cacodylate buffer. Bacterial sections were thawed and stained/embedded in a 4% uranyl acetate-2% methyl cellulose mixture (1:9). Images were recorded with a Tecnai Spirit 120 KV (with a bottom-mounted Eagle 4kx4k camera). Around 50 to 100 bacteria were scored under each condition.

### Phase-contrast microscopy.

Exponentially growing samples were prepared as described for SEM analysis and fixed with 2.5% glutaraldehyde in culture medium for 15 min at room temperature, followed by overnight fixation at 4°C. Soft-agar pads were prepared with 2% agarose solution in PBS (1×), and 10 μL of fixed bacteria was spotted on the agar pad and covered with a coverslip. Image acquisition was performed on an inverted phase-contrast Carl Zeiss Axio Observer Z1 microscope using a 100× objective. Quantification of bacteria and cell-length determination were done using Fiji software with a MicrobeJ plugin.

### Hi-C analysis.

**(i) Preparation of bacteria.** H. pylori strain G27 was grown with or without 2.5 μM bismuth in liquid Brucella medium as indicated above. One-hundred-milliliter cultures were set up, starting with a OD_600_ of 0.01, and incubated at 37°C for 16 h until a final OD of 0.50 corresponding to 10^9^ CFU was reached. Formaldehyde was added (3% final) to the bacterial culture, and the mixture was incubated under gentle agitation at room temperature for 30 min and then at 4°C for 30 min. Fifteen milliliters of glycine, 2.5 M, was then added, followed by an incubation at room temperature for 20 min under gentle agitation. The solution was centrifuged (6,000 × *g*, 10 min, 4°C), and the pellet was washed in 1× PBS. After another similar centrifugation, the supernatant was removed and the pellet was directly frozen at −80°C.

**(ii) Hi-C library generation.** Hi-C libraries were generated as previously described (65). The bacterial pellet was resuspended in 1.2 mL 1× TE supplemented with antiprotease (mini tablets; Roche), transferred into a Precellys tube (2 mL; VK05 supplemented with 100 μL of VK01 glass beads), and disrupted (6,700 rpm, 20 s on/30 s off, 6 cycles). Lysates were recovered, 10% SDS was added to a final concentration of 0.5%, and the lysate was incubated for 10 min at room temperature. For each library, 1 mL of lysate was transferred to a tube containing the digestion reaction solution (500 μL of NEB1 10× buffer, 500 μL of 10% Triton, 1,000 U of Sau3AI, and H_2_O, final volume of 4 mL). Digestion was allowed to proceed for 3 h at 37°C under gentle agitation. Tubes were then centrifuged for 20 min at 4°C and 16,000 × *g*, the supernatants were discarded, and pellets were resuspended in 400 μL H_2_O. Biotinylation was done by adding 50 μL of 10× NEB ligation buffer (without ATP), 4.5 μL of 10 mM dATP/dTTP/dGTP, 37.5 μL of biotin-dCTP (0.4 mM), and 8 μL of Klenow fragment (5 U/μL). Reaction mixtures were incubated for 45 min at 37°C and then transferred to a tube containing the ligation reaction mixture (160 μL of 10× NEB ligation buffer, 16 μL of 100 mM ATP, 16 μL of 10 mg/mL BSA, and 500 U of T4 DNA ligase; final volume of 1.1 mL). Ligation reaction mixtures were processed for 3 h at room temperature. Totals of 20 μL of 0.5 M EDTA, 80 μL of 10% SDS, and 2 mg of proteinase K were added to each reaction mixture and incubated overnight at 65°C to digest the proteins. DNA was extracted using phenol-chloroform and precipitated with 2.5 vol of 100% ethanol. Pellets were suspended in a final volume of 130 μL of 1× TE supplemented with RNase, incubated for 1 h at 37°C, and stored at −20°C until use. DNA was extracted, purified, and processed into the sequencing library as described previously (65). Proximity ligation libraries were sequenced using paired-end (PE) Illumina sequencing (2 × 35 bp; NextSeq 500 apparatus).

**(iii) Hi-C analysis.** Reads were aligned with bowtie2 v2.3.5.1 (66), and Hi-C matrices (5 kbp resolution) were generated using hicstuff v3.0.3 (https://github.com/koszullab/hicstuff). Balanced normalizations were performed using the ICE algorithm (67). Comparisons between matrices (10-kbp resolution) were done using a log_2_ ratio for flexible binning (68).

### Statistical analysis.

Statistical analyses of the size of bacteria and of purified MVs from bismuth-treated or control conditions were performed with the nonparametric Kolmogorov-Smirnov test. Statistical analyses were carried out by one-way analysis of variance (ANOVA) or by Mann-Whitney tests. GraphPad Prism 8 was used to perform the statistical analysis.

### Data availability.

All data needed to evaluate the conclusions in the paper are present in the paper and/or the supplemental material. Additional data related to this paper may be requested from the authors. Mass spectrometry proteomics data are available at the ProteomeXchange Consortium via the PRIDE partner repository with the data set identifier PXD028509.
