# Supplementary figures and images for: Bacterial Membrane Vesicles as a Novel Strategy for Extrusion of Antimicrobial Bismuth Drug in Helicobacter pylori
Source: mBio. 2022 Sep 26;13(5):e01633-22. doi: 10.1128/mbio.01633-22 (PMC9601102; doi:10.1128/mbio.01633-22)

Figure. S1

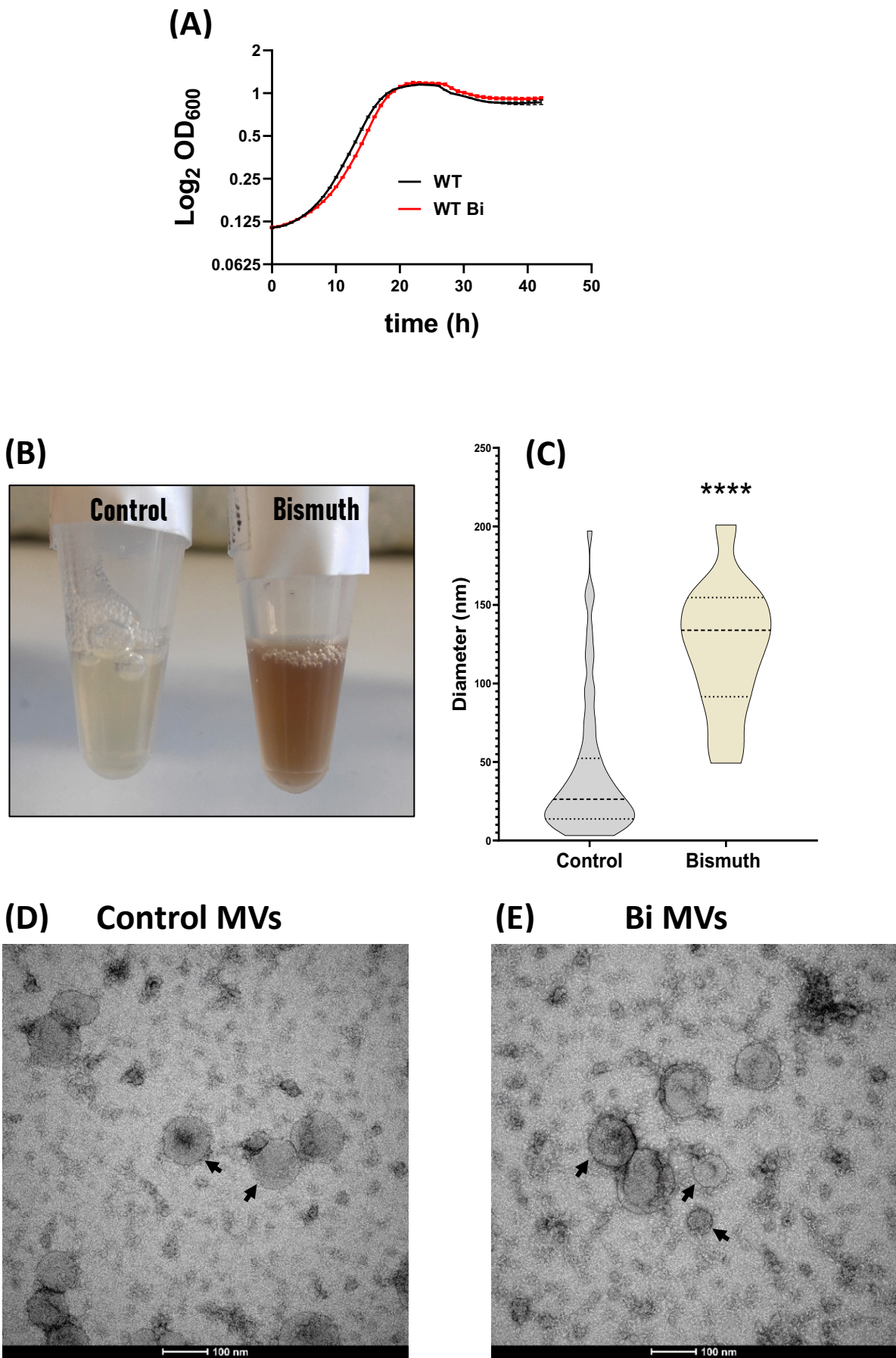

Supplement: FIG S1 [file mbio.01633-22-s0001.pdf]

Figure S2

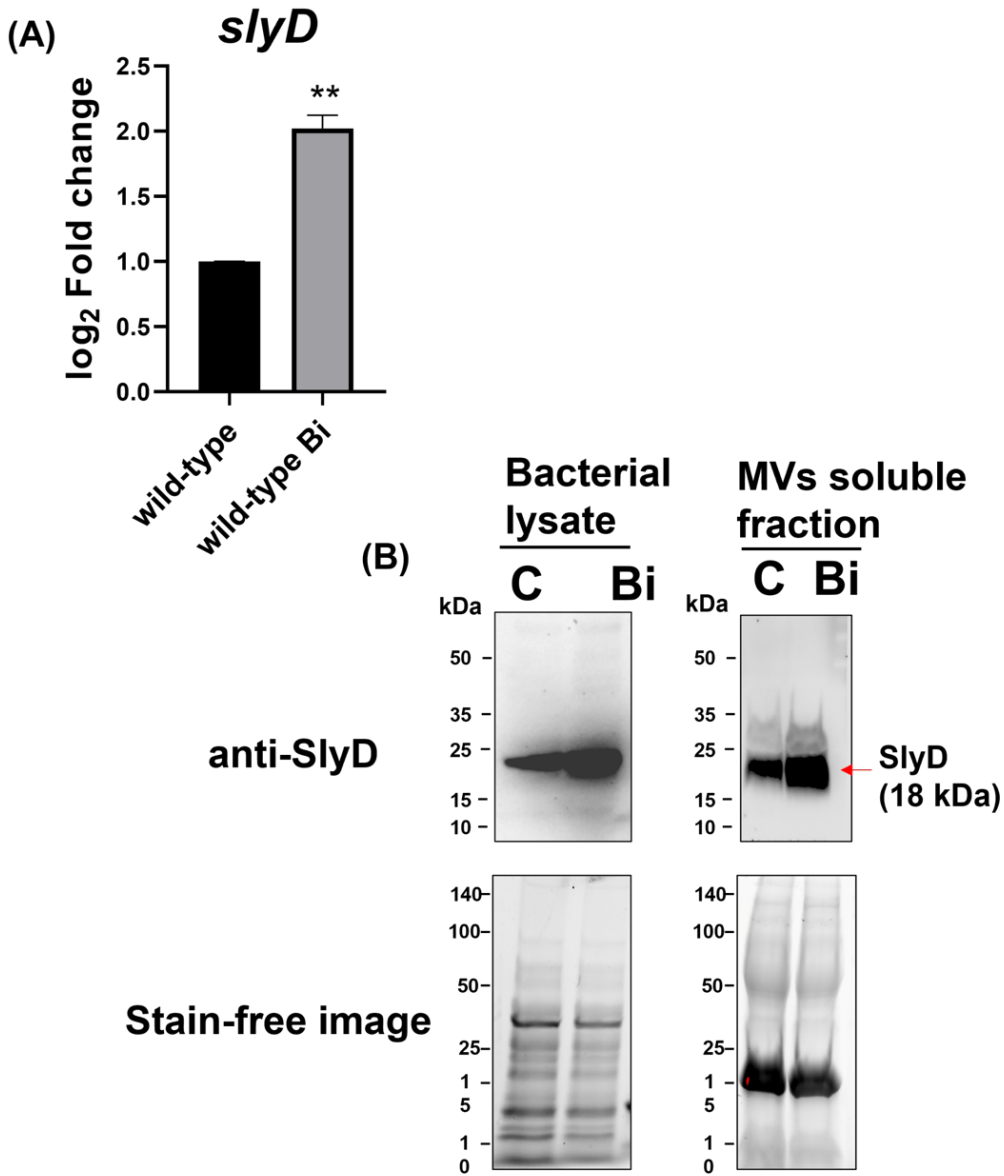

Supplement: FIG S2 [file mbio.01633-22-s0002.pdf]

Figure S3

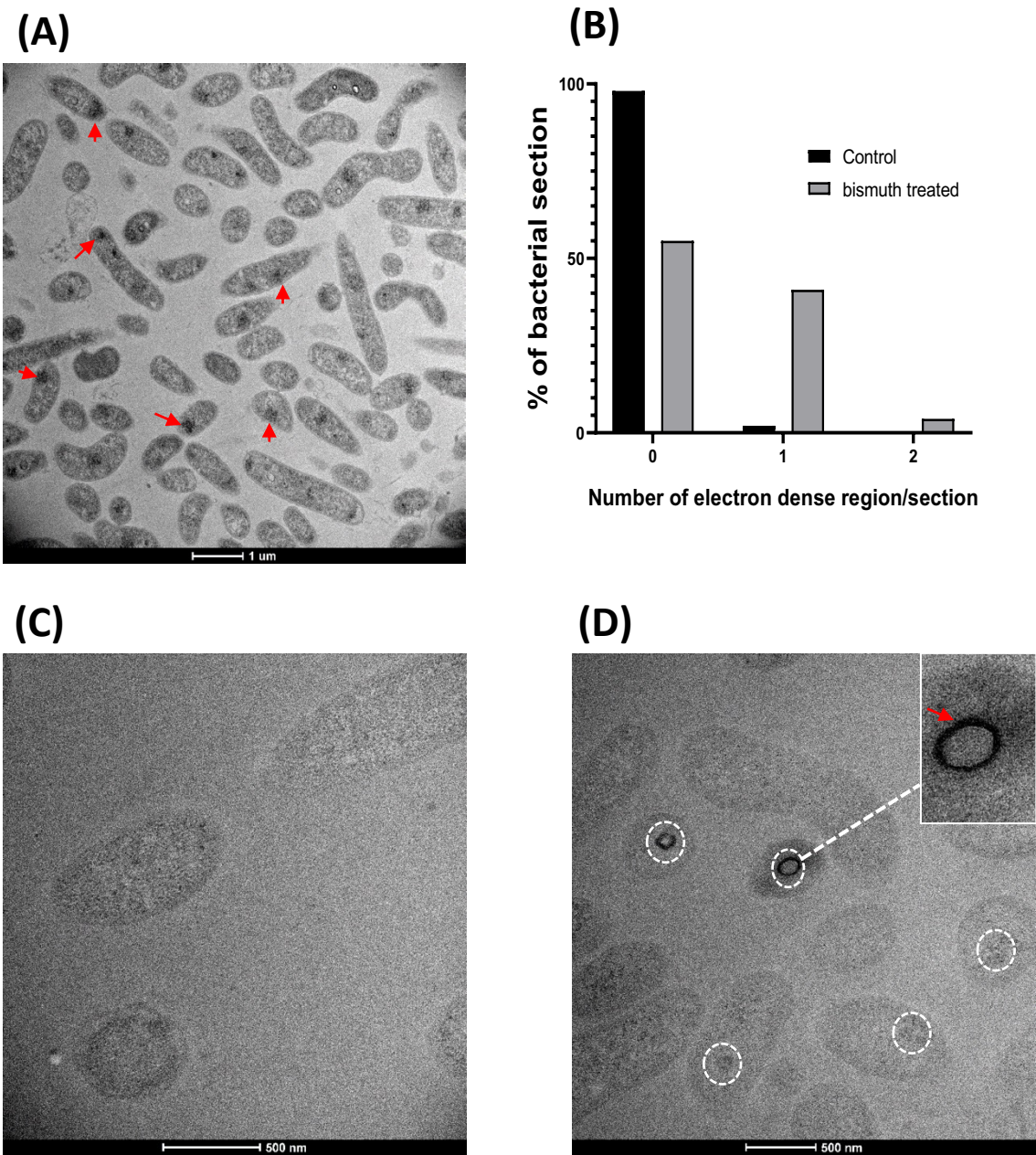

Supplement: FIG S3 [file mbio.01633-22-s0003.pdf]

Figure S4

(A)

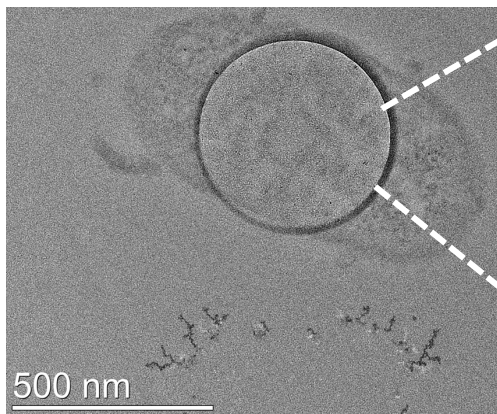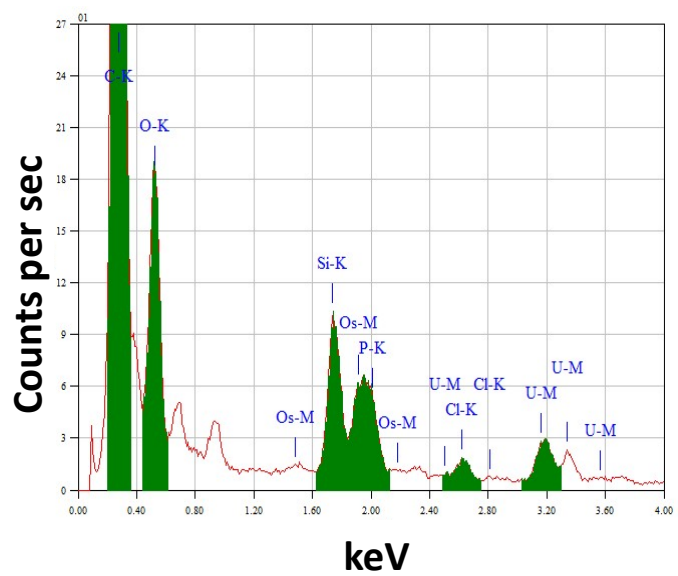

(B)

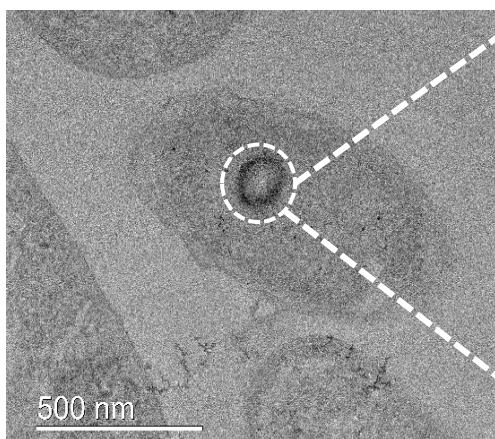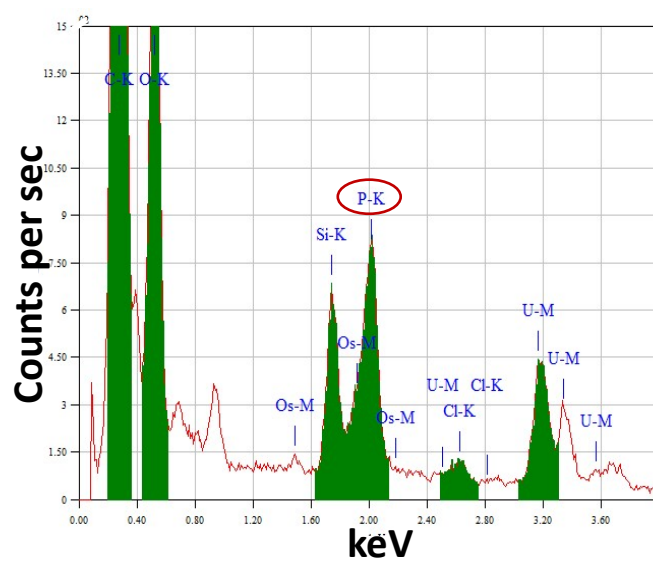

Supplement: FIG S4 [file mbio.01633-22-s0004.pdf]

Figure S5

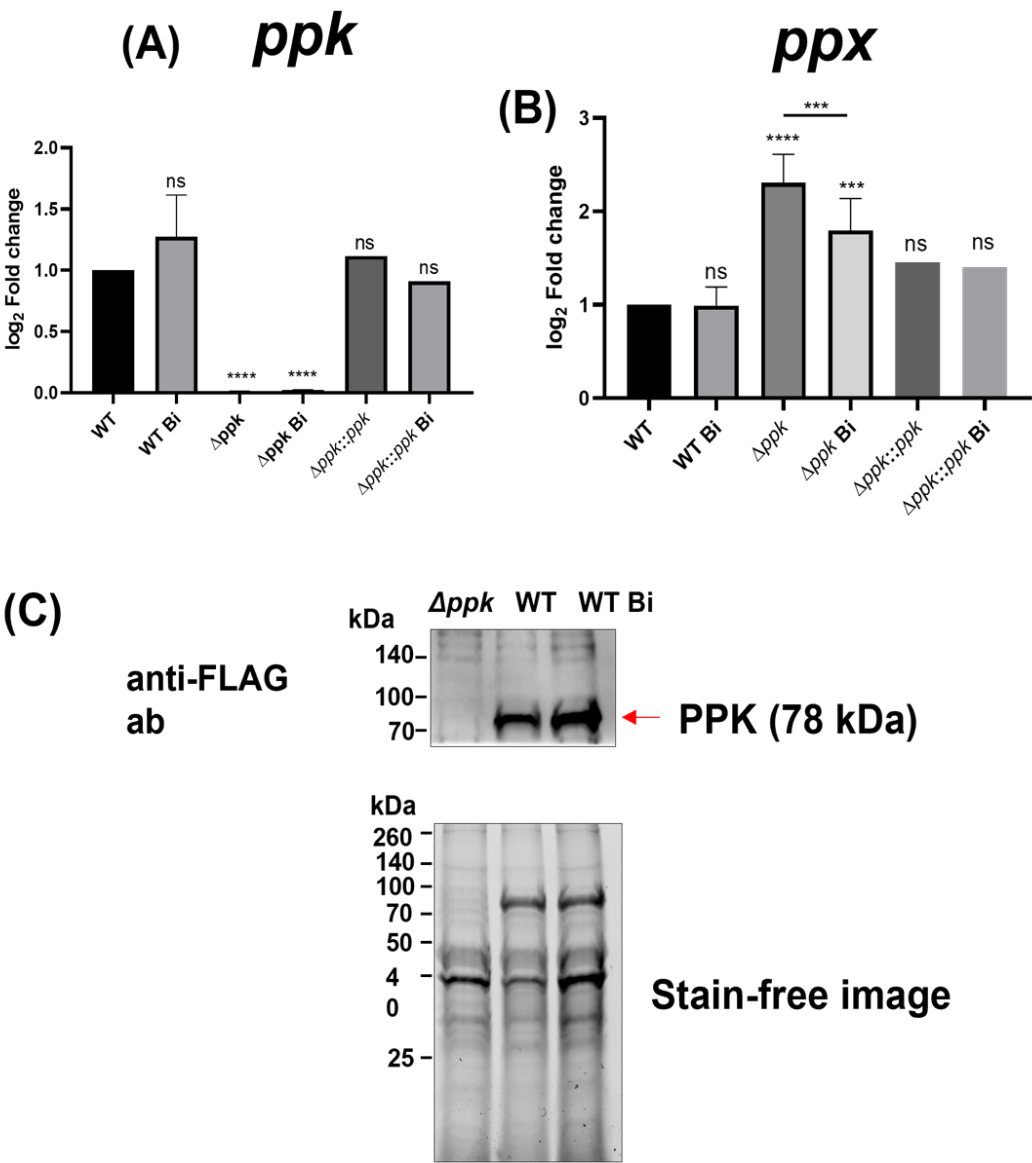

Supplement: FIG S5 [file mbio.01633-22-s0005.pdf]
