# Supplementary material for: Bacterial Membrane Vesicles as a Novel Strategy for Extrusion of Antimicrobial Bismuth Drug in Helicobacter pylori
Source: mBio. 2022 Sep 26;13(5):e01633-22. doi: 10.1128/mbio.01633-22 (PMC9601102; doi:10.1128/mbio.01633-22)
Supplement: TABLE S1 [file mbio.01633-22-s0006.docx]

**Table S1:**

**(A) Proteins present and absent in the MVs originated from bismuth treated bacteria compared to untreated bacteria.**

**List of proteins which were found to be uniquely present in vesicles purified from bismuth treated bacteria as compared to MVs from untreated bacteria.**

| **Gene name** | **Protein function** | **Mol. Weight (KDa)** |
| --- | --- | --- |
| ***HPG27_65** | **Urease accessory protein (UreE)** | 19.43 |
| HPG27_127 | L-lactate permease | 59.26 |
| ***HPG27_273** | **Aliphatic amidase (AmiE)** | 37.71 |
| HPG27_299 | Uncharacterized protein | 27.48 |
| HPG27_378 | Homoserine kinase (ThrB) | 32.66 |
| HPG27_398 | Flagellar motor switch protein (FliN) | 31.95 |
| HPG27_404 | Co-chaperone-curved DNA binding protein A (CbpA) | 32.97 |
| HPG27_468 | 4-hydroxy-tetrahydrodipicolinate reductase (DapB) | 27.82 |
| HPG27_478 | Beta-lactamase | 33.48 |
| HPG27_535 | Signal peptidase I (LepB) | 33.81 |
| HPG27_585 | Histidinol-phosphate transaminase | 43.15 |
| HPG27_618 | Cyclic dehypoxanthine futalosine synthase (MqnC) | 40.03 |
| HPG27_646 | Acetyl coenezyme A acetyltransferase | 41.22 |
| HPG27_712 | Molybdopterin biosynthesis protein (MoeB) | 25.56 |
| ***HPG27_784** | **Thioredoxin reductase (TrxB)** | 33.56 |
| HPG27_844 | Acyl coenzyme A thioesterase | 21.53 |
| ***HPG27_854** | **Hydrogenase nickel accessory protein (HypB)** | 27.35 |
| HPG27_870 | Glyceraldehyde 3-phosphate dehydrogenase (GapA) | 37.17 |
| HPG27_898 | Acetyl-CoA carboxylase carboxyl transferase subunit beta (AccD) | 32.55 |
| HPG27_904 | RNA pseudouridine synthase | 27.48 |
| ***HPG27_906** | **Uncharacterized protein** | 29.20 |
| HPG27_1166 | D-lactate dehydrogenase | 106.03 |
| HPG27_1194 | Shikimate dehydrogenase (AroE) | 29.08 |
| HPG27_1198 | Tryptophan-tRNA ligase (TrpS) | 36.82 |
| HPG27_1210 | NADH-ubiquinone oxidoreductase chain F | 36.77 |
| HPG27_1252 | 50s ribosomal proteins L18 (RplR) | 13.46 |
| HPG27_1255 | 30s ribosomal protein S14 (RpsZ) | 7.04 |
| HPG27_1261 | 50s ribosomal protein L16 (RplP) | 16.04 |
| HPG27_1320 | UDP-N-acetylglucosamine acyltransferase | 29.79 |
| HPG27_1363 | Peptidyl-prolyl cis-trans isomerase B | 17.57 |
| HPG27_1373 | tRNA modification GTPase (MnmE) | 45.15 |
| HPG27_1509 | Regulatory protein (DniR) | 42.90 |

*** Bi binding proteins identified in previous studies** (Wang et al. 2017)

**List of proteins uniquely absent in the vesicles purified from bismuth treated bacteria as compared to MVs from untreated bacteria.**

| **Gene name** | **Protein function** | **Mol. Weight (KDa)** |
| --- | --- | --- |
| HPG27_245 | Dihydroorotase (PyrC) | 42.04 |
| HPG27_248 | tRNA-2-methylthio-N(6)-dimethylallyladenosine synthase (MiaB) | 49.33 |
| HPG27_303 | PolyE-rich protein | 55.58 |
| HPG27_369 | DNA helicase (RuvB) | 37.32 |
| HPG27_517 | Acetyl=coenzyme A carboxylase carboxyl transferase subunit alpha (AccA) | 34.92 |
| HPG27_588 | Beta-lactamase | 38.06 |
| HPG27_748 | DNA transformation competence protein (ComM) | 57.31 |
| HPG27_789 | Glutamyl-tRNA amidotransferase subunit A (GatA) | 49.65 |
| HPG27_925 | Cell division protein (FtsA) | 55.18 |
| HPG27_976 | Uncharacterized protein | 75.77 |
| HPG27_1173 | Aspartokinase protein | 44.23 |
| HPG27_1177 | Uncharacterized protein | 17.33 |
| HPG27_1334 | Uncharacterized protein | 69.97 |
| HPG27_1385 | Putative secreted motility protein | 22.91 |

**(B) Differentially abundant proteins present in MVs of bismuth-treated and nontreated samples.**

**Proteins which were found to be more abundant in MVs of bismuth sample.** Bismuth-binding proteins are shown in bold.

| **Gene name** | **Protein function** | **Mol. Weight (KDa)** | **Log_2_ (Bi/C) fold change** | **Adjusted p-value** |
| --- | --- | --- | --- | --- |
| HPG27_1383 | DNA polymerase III subunit alpha | 137.95 | 4.76 | 0.003 |
| HPG27_1242 | 50S Ribosomal protein L17 (RplQ) | 13.39 | 4.74 | 0.000 |
| HPG27_217 | Porphobililinogen deaminase (HemC) | 33.95 | 4.40 | 0.005 |
| HPG27_115 | 50S Ribosomal protein L20 (RplT) | 14.01 | 3.89 | 0.001 |
| HPG27_600 | 7-cyano-7-deazaguanine synthase (QueC) | 25.33 | 3.84 | 0.001 |
| HPG27_1053 | Pyruvate ferredoxin oxidoreductase, beta subunit | 34.97 | 3.82 | 0.001 |
| HPG27_149 | Delta-aminolevulinic acid dehydratase | 36.2 | 3.80 | 0.000 |
| HPG27_228 | PHB domain-containing protein | 40.00 | 3.48 | 0.001 |
| ***HPG27_401** | **iron-responsive transcriptional regulator (Fur)** | 17.74 | 3.41 | 0.000 |
| HPG27_1020 | Thiol:disulfide interchange protein | 25.55 | 3.34 | 0.001 |
| HPG27_43 | agmatine deiminase | 37.45 | 3.22 | 0.006 |
| HPG27_108 | DNA topoisomerase 1 (TopA) | 83.26 | 2.83 | 0.000 |
| HPG27_1314 | Adenine DNA-methyltransferase | 55.54 | 2.79 | 0.005 |
| HPG27_632 | Aminotransferase | 42.80 | 2.76 | 0.000 |
| HPG27_1141 | 30S ribosomal protein S12 (RpsL) | 15.10 | 2.67 | 0.008 |
| ***HPG27_1069** | **Peptidyl-prolyl cis-trans isomerase (SlyD)** | 18.58 | 2.67 | 0.001 |
| HPG27_275 | 50S ribosomal protein L21 (RplU) | 11.88 | 2.66 | 0.001 |
| HPG27_1345 | Isoleucine--tRNA ligase (IleS) | 106.1 | 2.61 | 0.000 |
| HPG27_605 | Glutamate--tRNA ligase 2 (GltX2) | 51.08 | 2.59 | 0.004 |
| HPG27_577 | Aspartate--tRNA(Asp/Asn) ligase (AspS) | 65.33 | 2.50 | 0.004 |
| HPG27_1097 | Valine--tRNA ligase (ValS) | 101.37 | 2.49 | 0.009 |
| ***HPG27_10** | **Heat shock protein A (HspA)** | 12.98 | 2.45 | 0.006 |
| HPG27_1140 | 30S ribosomal protein S7 (RpsG) | 16.60 | 2.45 | 0.005 |
| HPG27_218 | Proline--tRNA ligase (ProS) | 65.1 | 2.33 | 0.000 |
| HPG27_257 | Guanosine pentaphosphate phosphohydrolase | 54.29 | 2.32 | 0.001 |
| HPG27_1497 | Flagellar basal body rod protein (FlgB) | 15.80 | 2.29 | 0.001 |
| HPG27_1091 | 50S ribosomal protein L19 (RplS) | 13.63 | 2.29 | 0.003 |
| HPG27_1253 | 50S ribosomal protein L6 (RplF) | 19.47 | 2.24 | 0.007 |
| HPG27_647 | Succinyl-CoA-transferase subunit A | 25.35 | 2.23 | 0.008 |
| HPG27_1267 | 50S ribosomal protein L4 (RplD) | 24.09 | 2.21 | 0.006 |
| HPG27_132 | Cytochrome coxidase monoheme subunit | 26.52 | 2.15 | 0.006 |
| HPG27_1129 | Alpha-carbonic anhydrase | 28.32 | 2.15 | 0.005 |
| HPG27_170 | DUF1882 domain-containing protein | 21.20 | 2.05 | 0.002 |
| HPG27_1263 | 50S ribosomal protein L22 (RplV) | 13.10 | 1.99 | 0.004 |
| ***HPG27_176** | **Fumarate reductase (FrdA)** | 80.15 | 1.98 | 0.001 |
| HPG27_1211 | NADH-ubiquinone oxidoreductase chain G | 95.24 | 1.94 | 0.002 |
| HPG27_812 | Bifunctional protein (HldE) | 50.91 | 1.92 | 0.009 |
| HPG27_730 | NMO domain-containing protein | 39.59 | 1.90 | 0.002 |
| HPG27_1519 | Pyridoxine 5-phosphate synthase (PdxJ) | 29.72 | 1.82 | 0.002 |
| HPG27_994 | Phenylalanine--tRNA ligase alpha subunit (PheS) | 38.04 | 1.81 | 0.003 |
| HPG27_1262 | 30S ribosomal protein S3 (RpsC) | 26.46 | 1.78 | 0.005 |
| HPG27_227 | ATP-dependent RNA helicase (RhpA) | 55.77 | 1.77 | 0.005 |
| HPG27_1244 | 30S ribosomal protein S4 (RpsD) | 23.97 | 1.73 | 0.003 |
| HPG27_33 | PDZ domain-containing protein | 38.24 | 1.73 | 0.004 |
| HPG27_1143 | DNA-directed RNA polymerase, beta subunit | 7.69 | 1.70 | 0.000 |
| HPG27_1258 | 50S ribosomal protein L14 (RplN) | 13.28 | 1.67 | 0.004 |
| HPG27_529 | Aminopeptidase (PepA) | 54.50 | 1.67 | 0.001 |
| HPG27_77 | 50S ribosomal protein L13 (RplM) | 16.14 | 1.66 | 0.004 |
| HPG27_1025 | dCTP deaminase (DcD) | 20.88 | 1.64 | 0.003 |
| HPG27_1419 | 50S ribosomal protein L25 (RplY) | 19.91 | 1.63 | 0.004 |
| HPG27_1384 | Cytochrome c551 peroxidase | 38.69 | 1.62 | 0.003 |
| HPG27_1268 | 50S ribosomal protein L3 (RplC) | 21.19 | 1.61 | 0.001 |
| HPG27_992 | NifS-like protein | 48.62 | 1.53 | 0.005 |
| HPG27_79 | malate:quinone oxidoreductase (MqO) | 50.50 | 1.50 | 0.006 |
| HPG27_1042 | Glucose-6-phosphate 1-dehydrogenase | 49.30 | 1.46 | 0.002 |
| HPG27_113 | translation initiation factor IF-3 | 23.33 | 1.46 | 0.009 |
| HPG27_1181 | Carbamoyl-phosphate synthase small chain (CarA) | 41.69 | 1.45 | 0.008 |
| HPG27_520 | 3-oxoacyl-[acyl-carrier-protein] reductase | 26.75 | 1.44 | 0.008 |
| HPG27_81 | RNA polymerase sigma factor (RpoD) | 79.09 | 1.41 | 0.004 |
| HPG27_467 | Glycolate oxidase subunit (GlcD) | 50.53 | 1.40 | 0.001 |
| HPG27_627 | Coproporphyrinogen-III oxidase | 53.40 | 1.38 | 0.003 |
| HPG27_189 | Iron-sulfur cluster carrier protein (Mrp) | 39.90 | 1.35 | 0.001 |
| HPG27_1096 | Signal recognition particle protein (FfH) | 49.13 | 1.32 | 0.003 |
| HPG27_23 | Citrate synthase (GltA) | 48.37 | 1.27 | 0.006 |
| HPG27_1265 | 50S ribosomal protein L2 (RplB) | 30.27 | 1.27 | 0.003 |
| HPG27_995 | Phenylalanine--tRNA ligase beta subunit (PheT) | 85.00 | 1.26 | 0.002 |
| HPG27_205 | methionine sulfoxide reductase (MsrA) | 41.23 | 1.25 | 0.005 |
| HPG27_444 | Catalase-related peroxidase | 35.90 | 1.25 | 0.008 |
| HPG27_2 | Fido domain-containing protein | 28.10 | 1.24 | 0.004 |
| HPG27_1045 | Mannitol dehydrogenase | 38.57 | 1.23 | 0.001 |
| HPG27_408 | Response regulator | 35.17 | 1.23 | 0.006 |
| ***HPG27_9** | **60 kDa chaperonin (GroEL)** | 58.27 | 1.18 | 0.003 |
| HPG27_616 | Aminodeoxyfutalosine synthase (MqnE) | 41.36 | 1.18 | 0.002 |
| HPG27_1224 | Tryptophan synthase beta chain (TrpB) | 42.74 | 1.18 | 0.002 |
| HPG27_1254 | 30S ribosomal protein S8 (RpsH) | 15.18 | 1.17 | 0.006 |
| HPG27_1237 | Acid phosphatase lipoprotein | 26.31 | 1.16 | 0.000 |
| HPG27_391 | Proline peptidase | 40.69 | 1.15 | 0.006 |
| HPG27_510 | Transcription termination factor (Rho) | 49.54 | 1.15 | 0.006 |
| HPG27_839 | Cysteine--tRNA ligase (CysS) | 53.19 | 1.13 | 0.008 |
| HPG27_878 | Geranyltrans transferase | 34.22 | 1.08 | 0.008 |
| HPG27_989 | Uncharacterized protein | 18.67 | 1.05 | 0.004 |
| HPG27_1256 | 50S ribosomal protein L5 (RplE) | 20.27 | 1.04 | 0.006 |
| HPG27_1293 | Phosphoglycerate kinase (pgk) | 44.69 | 1.04 | 0.007 |
| HPG27_6 | Pantothenate synthetase (PanC) | 31.27 | 1.03 | 0.003 |
| HPG27_99 | Cysteine synthetase (CysK) | 32.95 | 1.02 | 0.009 |
| HPG27_446 | Outer membrane protein (HofD) | 53.58 | 1.02 | 0.005 |
| HPG27_791 | Polyamine aminopropyltransferase (SpeE) | 30.51 | 1.00 | 0.003 |

***Previously identified bismuth-binding proteins (Wang et al. 2017).**

**Proteins which were found to be less abundant in MV of bismuth treated sample.**

| **Gene name** | **Protein function** | **Mol. Weight (KDa)** | **Log_2_ (Bi/C) fold change** | **Adjusted p-value** |
| --- | --- | --- | --- | --- |
| HPG27_747 | Cadmium-transporting ATPase (CadA) | 74.84 | -7.68 | 0.000 |
| HPG27_1159 | Polyribonucleotide nucleotidyltransferase (PNP) | 77.16 | -4.84 | 0.000 |
| HPG27_558 | 8-amino-7-oxononanoate synthase (BioF) | 43.19 | -3.02 | 0.003 |
| HPG27_924 | Peptidyl-prolylcis-transisomerase D | 58.76 | -2.93 | 0.000 |
| HPG27_83 | Malonyl CoA-acyl carrier protein transacylase | 34.38 | -2.43 | 0.000 |
| HPG27_1163 | Phosphoribosylamine--glycine ligase (PurD) | 47.42 | -2.14 | 0.003 |
| HPG27_1006 | CheA-MCP coupling protein (CheW) | 19.00 | -2.05 | 0.003 |
| PG27_576 | Chemotaxis protein (CheV2) | 35.63 | -2.00 | 0.003 |
| HPG27_1202 | Orotate phosphoribosyltransferase (PyrE) | 21.96 | -1.92 | 0.006 |
| HPG27_1149 | Transcription termination/antitermination protein (NusG) | 20.26 | -1.95 | 0.007 |
| HPG27_277 | heme binding protein (HbpA) | 62.35 | -1.45 | 0.008 |
| HPG27_283 | Alginate_lyase domain-containing protein | 37.69 | -1.38 | 0.005 |
| HPG27_5 | Orotidine 5-phosphate decarboxylase | 25.26 | -1.36 | 0.003 |
| HPG27_866 | Iron-regulated outer membrane protein (FrpB2) | 90.45 | -1.08 | 0.003 |

Wang, Y. *et al.* (2017) Integrative approach for the analysis of the proteome-wide response to bismuth drugs in *Helicobacter pylori*, Chemical Science, 8(6), pp. 4626–4633. doi:10.1039/C7SC00766C.
