# Supplementary material for: Bacterial Membrane Vesicles as a Novel Strategy for Extrusion of Antimicrobial Bismuth Drug in Helicobacter pylori
Source: mBio. 2022 Sep 26;13(5):e01633-22. doi: 10.1128/mbio.01633-22 (PMC9601102; doi:10.1128/mbio.01633-22)
Supplement: TABLE S2 [file mbio.01633-22-s0007.docx]

**Table S2: Measurement of Bismuth levels in *H. pylori* cellular lysates and purified MVs.** Bismuth concentration was determined by measuring the emission spectra at three different wavelengths. Subscript values under λ corresponds the emission signal observed at different wavelengths (mean average of biological and technical replicates). The limit of detection is 10 µg/L (10 ppb). Mass ratio (%) was calculated by dividing the mass of bismuth detected in the sample by total mass of the sample used for analysis. WT: wild type.

| **Sample** | **Bismuth concentration (µg/L)** | | | **Mass ratio (%)** |
| --- | --- | --- | --- | --- |
|  | **λ_222.821_** | **λ_223.061_** | **λ_306.771_** |  |
| G27 (WT) | 10.913 | -0.334 | -0.481 | 0.000 |
| G27 + Bi 2.5 µM | 15.543 | 10.262 | 10.361 | 0.001 |
| MVs of WT | 6.814 | -0.848 | -1.568 | 0.000 |
| MVs of WT + Bi 2.5 µM | 688.217 | 683.052 | 676.287 | 0.392±0.18 |
| G27Δ*ppk* | 1.3269 | -3.8541 | -14.4872 | -0.0008 |
| G27*Δppk* + Bi 2.5 µM | 11.257 | 8.3753 | -4.113 | 0.0008 |
| MVs of Δ*ppk* | 16,7257 | 9,1201 | -3,03302 | 0.001 |
| MVs of *Δppk* + Bi 2.5 µM | 818.95 | 895.047 | 912.70 | 1.083±0.59 |
