# Supplementary material for: Bacterial Membrane Vesicles as a Novel Strategy for Extrusion of Antimicrobial Bismuth Drug in Helicobacter pylori
Source: mBio. 2022 Sep 26;13(5):e01633-22. doi: 10.1128/mbio.01633-22 (PMC9601102; doi:10.1128/mbio.01633-22)
Supplement: TABLE S3 [file mbio.01633-22-s0008.docx]

**Table S3:**

**A) List of strains used in the study**

| **Strains** | **Relevant characteristics** | **Reference** |
| --- | --- | --- |
| *Helicobacter pylori* | | |
| G27 | Sequenced parental strain | Baltrus *et al*., 2009 |
| G27 ∆*ppk* kan^R^ | *ppk* deletion mutant | this work |
| G27 ∆*ppk*::*ppk* apra^R^ | *ppk* complemented mutant | this work |
| G27 *ppk*-FLAG kan^R^ | FLAG-tag fusion of the *ppk* gene | this work |

**B) List of primers used in the study**

| **Name** | **Sequence 5’-3’** |
| --- | --- |
| ppk D1 | GTTACCCCCAACCCTAACATAC |
| ppk D2 | CTCATTTTAGCCATTTATTCCTCTAGAAATCCTTTAAATCATCATG |
| ppk D3 | GAATTGTTTTAGTACCTGGAGGGAATAAGGTTATCGTTCAAATAATTAAAAG |
| ppk D4 | ATTAAACCCCATCGCATTTTG |
| ppk qF | GTTACCCCCAACCCTAACATAC |
| ppk qR | ATTAAACCCCATCGCATTTTG |
| ppx qF | AGGGCGTTGAGTAAAGTGCT |
| ppx qR | CGCCCCACTCCTAATGCTAT |
| Kan F | GAGGAATAAATGGCTAAAATGAGAATATC |
| Kan R | TATTCCCTCCAGGTACTAAAACAATTC |
| Apra F | GATTACAAGGATGACGACGATAAGTAAGGAGTGCAATGTCGTGCAATAC |
| Apra R | TATTCCCTCCAGGTATCAGCCAATCGACTGGC |
| ppk C1 | GTTACCCCCAACCCTAACATAC |
| ppk C2 | TTACTTATCGTCGTCATCCTTGTAATCAAAGGTTTTAAGGGCTTGTTTTTCAA |
| ppk C3 | GTCGATTGGCTGATACCTGGAGGGAATAAGGTTATCGTTCAAATAATTAAAAGAC |
| ppk C4 | CTTGCTCTATGTGTTTGTTTTTGCC |

Baltrus, D.A. *et al.* (2009) The complete genome sequence of *Helicobacter pylori* strain G27, *Journal of Bacteriology*, 191(1), pp. 447–448. doi:10.1128/JB.01416-08.
